# Immunomarker profiling in human chronic wound swabs reveals IL-1 beta/IL-1RA and CXCL8/CXCL10 ratios as potential biomarkers for wound healing, infection status and regenerative stage

**DOI:** 10.1186/s12967-025-06417-2

**Published:** 2025-04-08

**Authors:** Julian-Dario Rembe, Waseem Garabet, Matthias Augustin, Joachim Dissemond, Wiebke Ibing, Hubert Schelzig, Ewa K. Stuermer

**Affiliations:** 1 Department for Vascular and Endovascular Surgery, University Hospital Duesseldorf (UKD), Heinrich Heine University Duesseldorf, Moorenstrasse 5, 40225 Duesseldorf, Germany; 2 Institute for Health Services Research in Dermatology and Nursing Professions (IVDP), University Medical Center Hamburg-Eppendorf (UKE), Hamburg, Germany; 3 Department of Dermatology, Venereology and Allergology, Essen University Hospital, Essen, Germany; 4 Clinic and Polyclinic for Vascular Medicine, University Heart and Vascular Center, University Medical Center Hamburg-Eppendorf (UKE), Hamburg, Germany

**Keywords:** Wound healing, Tissue regeneration, Immunomarker, Wound infection, Wound swabbing, Wound micro-environment, Immunoassay

## Abstract

**Background:**

Chronic wounds, such as diabetic foot ulcers, venous leg ulcers, and post-surgical wound healing disorders pose a significant challenge due to prolonged healing, risk of infection, and impaired quality of life. Persistent inflammation and impaired tissue remodeling are common in these wounds. Traditional diagnostic methods, including visual inspection and microbiological cultures, offer limited insight into the wound micro-environment. Immunomarker profiling could provide a deeper understanding of the molecular mechanisms underpinning wound healing, offering potential biomarkers for infection status and healing progression.

**Methods:**

This observational, multi-center cohort study, part of the ‘Wound-BIOME’ project, analyzed 110 swab samples from patients with acute and chronic wounds using multiplex immunoassays. Clinical parameters such as wound type, healing status, regeneration stage, and microbial burden were recorded. Total protein concentration was assessed, and 35 key immunomarkers, including cytokines (e.g. IL- 1α, IL- 1β), chemokines (CCL2, CXCL8, CXCL10), growth factors (FGF- 2, VEGF) and matrix metalloproteinases (MMP- 7, MMP- 9, MMP- 13), were quantified. Statistical analyses were performed to correlate immunomarker levels with clinical outcomes.

**Results:**

Pro-inflammatory markers, such as IL- 1β, IL- 18 and chemokines like CCL2 and CXCL8, were significantly elevated in non-healing and infected wounds compared to healing wounds. The study identified two new immunomarker ratios – IL- 1β/IL- 1RA and CXCL8/CXCL10 – as potential predictors of wound healing status. The IL- 1β/IL- 1RA ratio showed the highest accuracy for distinguishing healing from non-healing wounds (AUC = 0.6837), while the CXCL8/CXCL10 ratio was most effective in identifying infection (AUC = 0.7669).

**Conclusions:**

Immunomarker profiling via wound swabbing offers valuable insights into the wound healing process. Elevated levels of pro-inflammatory cytokines and MMPs are associated with chronic inflammation and impaired healing. The IL- 1β/IL- 1RA and CXCL8/CXCL10 ratios emerge as promising biomarkers to distinguish between infection and inflammation, with potential in targeted wound care. Further studies are needed to validate these findings and implement them in clinical practice.

**Supplementary Information:**

The online version contains supplementary material available at 10.1186/s12967-025-06417-2.

## Introduction

Chronic wounds, including diabetic foot ulcers (DFU), venous leg ulcers (VLU), and postsurgical wound healing disorders (WHD), represent a significant challenge due to prolonged healing times, susceptibility to infection, and impact on patient quality of life (QoL) as well as resources of the healthcare system [1, 2]. A persistent inflammatory state, impaired cellular proliferation, and defective remodeling processes are common in these wounds [3, 4]. In contrast, acute wound healing typically progresses through a well-coordinated sequence of healing phases of hemostasis, inflammation, proliferation, and remodeling, culminating in complete tissue repair [5]. Conventional diagnostic and monitoring methodologies for chronic wounds frequently rely on visual inspection, clinical evaluation, and microbiological cultures. These approaches, however, do not provide comprehensive data regarding the wound microenvironment and the progression of healing [6]. Understanding the molecular principles that differentiate chronic from acute wounds and healing from non-healing wounds is critical for developing targeted therapeutic strategies [7–9].

Recent advances in immunology and molecular biology have highlighted the pivotal role of various immunomarkers in wound healing. Interleukins, chemokines, matrix metalloproteinases (MMPs), and growth factors orchestrate the complex cellular and molecular events in wound repair. IL- 1β and IL- 6, for example, are key modulators of the inflammatory response. CCL2 and CXCL8, regulate leukocyte recruitment to the wound site. MMPs, such as MMP- 9, facilitate extracellular matrix (ECM) remodeling, while growth factors like Vascular Endothelial Growth Factor (VEGF) and Platelet-derived Growth Factor (PDGF) promote angiogenesis and fibroblast proliferation [5]. Aberrant expression of these markers is often observed in chronic wounds, contributing to their pathophysiology [4].

Multiplex immunoassay technologies have revolutionized the ability to simultaneously quantify multiple immunomarkers in clinical samples. This high-throughput approach allows for comprehensive profiling of the wound microenvironment, offering insights into the dynamic interplay of cytokines, chemokines, MMPs, and growth factors [10, 11]. Utilizing wound swabs as a minimally invasive sampling method, multiplex immunoassays enable real-time monitoring of the wound status, facilitating early detection of non-healing wounds and providing a basis for personalized interventions [10, 12, 13].

Current research underscores the potential of immunomarker profiling in enhancing chronic wound diagnostics and therapy monitoring [7, 11, 12, 14]. For instance, elevated levels of pro-inflammatory cytokines and proteases are indicative of chronic inflammation and matrix degradation *in-vitro* and in animal models, respectively, while deficiencies in specific growth factors can signal impaired healing [4, 5, 15–18]. By identifying these molecular signatures, clinicians could stratify patients based on wound pathology, tailor treatments to modulate specific pathways, and monitor therapeutic response through immunomarker measurements.

In this study, we aim to analyze a broad variety of immunomarkers, encompassing interleukins, chemokines, MMPs, and growth factors in patients with acute and chronic wounds using multiplex immunoassay and correlate findings with relevant clinical outcome parameters. Through this comprehensive approach, we seek to delineate the immunological landscape of wound regeneration, identify key biomarkers associated with healing status, and explore their utility in improving diagnostic accuracy and therapeutic outcomes. Our findings have the potential to inform the development of biomarker-driven wound management protocols, ultimately enhancing patient care, clinical outcomes and QoL in wound healing.

## Methods

### Study design and recruitment

The study is part of the'Wound-BIOME'project ("Breakdown, Identification and Observation of the Micro-Environment in acute and chronic Wounds"), a multicenter project with the aim of developing a better understanding of the biomolecular signatures in wound repair [13].

**Fig. 1:**
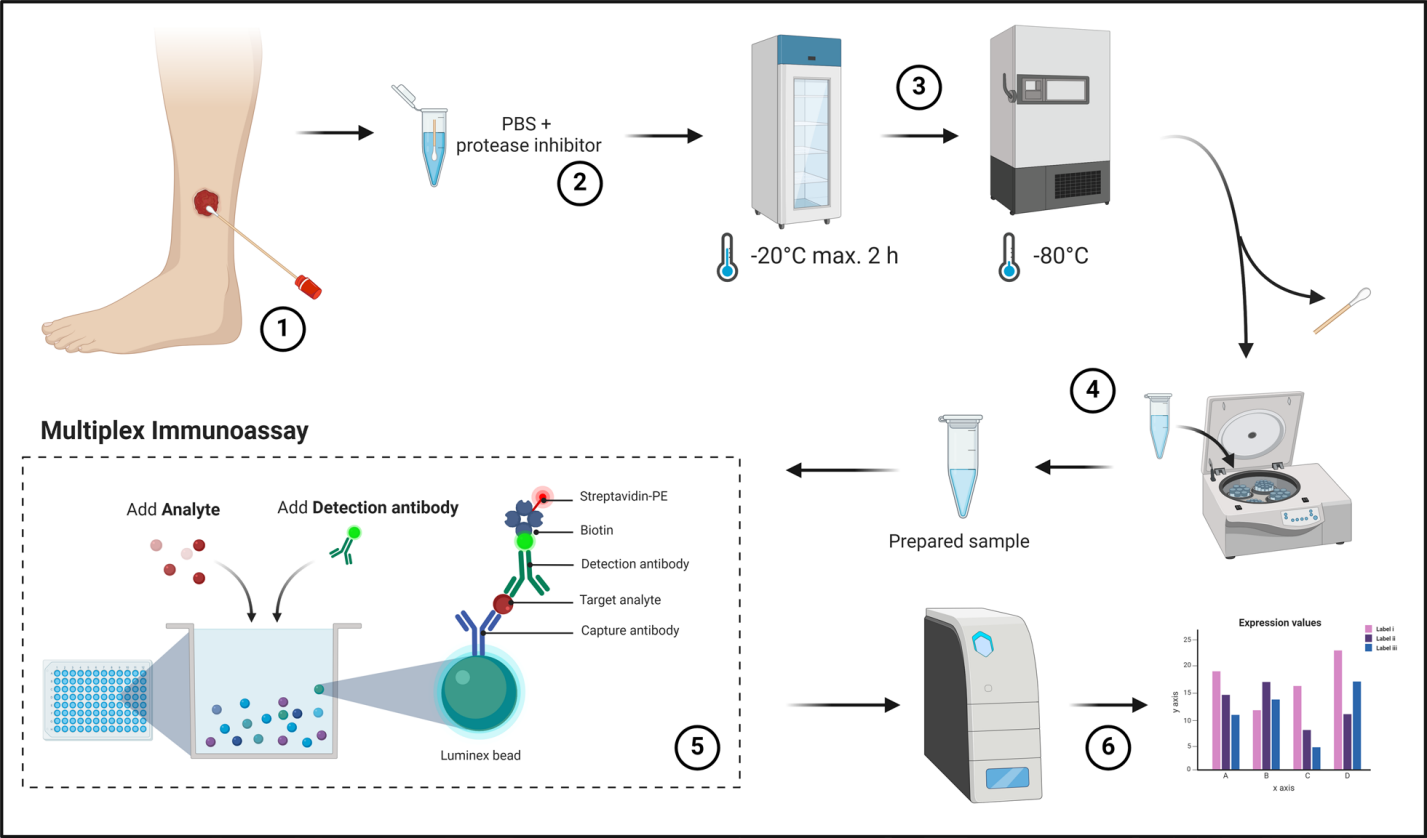
Sample acquisition and analysis process. The diagram shows the sample acquisition, preparation and analysis process. Wounds are sampled by swabbing using FLOQSwab swabs (1) according to the “Essener Rotary”. Samples are transported in a prepared transport media (2) and frozen within 2 h at a minimum of − 20°C and within 8 h at a maximum of − 80°C (3). Subsequently, sample processing is performed at a later time-point with removal of the swab, pelletizing of cellular and debris components and aliquoting of the sample material for further analysis (4). The prepared sample is subsequently analyzed using a multiplex immunoassay approach (5) and measured on the Luminex MAGPIX® platform for analyte quantification (6)

Figure 1 depicts the workflow for the immunomarker analysis using multiplex immunoassay. The study design is an observational, non-interventional, multi-center prospective cohort study. In addition to human biomaterial, clinical data including age, gender, wound area, wound entity and wound age was recorded. Sampled wounds were assessed regarding potential predictive outcomes or current stages by the responsible healthcare professional. Variables assessed were healing status (healing/non-healing), current wound healing stage (infection, inflammation, proliferation, epithelization) and microbial burden (non-infected, colonized, infected). The evaluation was strictly clinical, based on the wounds healing trajectory at the time of sampling and assessed by the primary wound care professional. These variables enable the correlation of clinical assessment with biomolecular findings. Supplementary Table 1 shows the complete set of variables evaluated for all investigated patients. Inclusion and exclusion criteria for the ‘Wound-BIOME’ cohort are given in Table 1 and the standardized sampling procedure was described in detail elsewhere [13].

**Table 1:** Inclusion and exclusion criteria of the Wound-BIOME project

Inclusion criteria	Exclusion criteria
• Patient ≥ 18 years	• Patient < 18 years
• Wound area ≥ 1.5 cm^2^	• Wound area < 1.5 cm^2^
• Presence of a wound which either - **Persists for more than 8 weeks** under adequate therapy ("**chronic**") **OR** is considered chronic due to their underlying disease* - **No shorter** than 24 h and **no longer** than 6 days after traumatic event/surgical intervention ("**acute**")	• Dry necrosis
	• Pregnancy and breastfeeding
	• Malignant genesis of the wound (e.g. ulcerating soft tissue tumor)

*Based on[19]

Seven different wound entities were included – acute wounds (AW), post-surgical wound-healing disorder (WHD), arterial leg ulcer (ALU), venous leg ulcer (VLU), diabetic foot ulcer (DFU), mixed arterio-venous ulcer (MIX) and pyoderma gangrenosum (PG). The differentiation between acute and chronic wounds was based on the national definition as reported in the standards of the German professional society “Initiative Chronische Wunden e.V. (ICW)” [19].

An ethics vote was obtained in advance for the ‘Wound-BIOME’ project. The leading positive ethics vote was issued by the Ethics Committee of the Private University of Witten/Herdecke (No. 11/2018). Further study centers followed with respective votes from the responsible ethics committees Essen (No. 18–8432-BO), Hamburg (No. PV5883) and Duesseldorf (No. 2020–1012). All recruited patients were informed in detail in advance about the study objectives, including data protection aspects, and written informed consent was obtained.

### Sample collection

Samples were collected from acute and chronic wounds via wound swabbing methodology using FLOQSwabs® (Copan Diagnostics Inc., Murrieta, USA). The specific process of sample collection and its utility has been described in detail in an earlier publication [13]. After obtaining the wound swab using the “Essen Rotary” technique [20], it was placed in a sample container with transport medium. Standard Eppendorf tubes (1.5 ml; Eppendorf SE, Hamburg, Germany) were used as sample containers, filled with 1 ml of phosphate-buffered saline (PBS) with a universal protease/phosphatase inhibitor (PPC1010; Sigma-Aldrich, Merck KGaA, Darmstadt, Germany) for protection from protein degradation.

The collected sample was frozen to at least − 18°C within two hours and to − 80°C within 24 h. Subsequent sample transport and handling was performed on ice. Excessive freeze/thaw cycles were avoided to minimize the risk of protein instability and degradation.

### Sample preparation for immunoassay analysis

One thawing cycle was necessary for sample preparation, whereby the swab tip was removed from the sample and coarse cellular and detrital components were pelletized via centrifugation. A sample aliquot of 210 µl was extracted for total protein quantification and subsequent multiplex immunoassays. Samples were carefully thawed on ice (2–8 °C) over a 2-h period. With the swab tip remaining in the tube samples were vortexed three times for 5 s to achieve the highest possible release of analytes into the transport medium. The tip was subsequently removed using sterile tweezers and rolled out once more on the inside of the sample vial. The swab tip was then discarded, and the sample again vortexed for 5 s for homogenization. This was followed by two centrifugation steps with 10,000xg for 15 min at 4 °C and transferring the supernatant to a new sample tube between the centrifugation steps to remove cellular and microbial detritus. The supernatant was then aliquoted and used for further investigations.

### Total protein quantification

Total protein content per sample was quantified using a colorimetric detection method based on Coomassie Brilliant Blue (Bradford assay). The analyses were conducted in accordance with the manufacturer's instructions (Coomassie (Bradford) Protein Assay Kit, Thermo Fisher Scientific Inc., Waltham, USA) and the results obtained using a spectrophotometer (EON™; BioTek Germany, Bad Friedrichshall, Germany).

### Multiplex immunoassay

A multiplex immunoassay was used to determine the concentration of 35 analytes including cytokines, chemokines, proteases, and growth factors. The Luminex MAGPIX system with xMAP® technology (DiaSorin S.p.A., Saluggia, Italy) was used in conjunction with specifically compiled Mix&Match ProcartaPlex™ Multiplex Immunoassay Kits (Thermo Scientific™, Waltham, USA). Sample preparation and kits were performed in strict accordance with the manufacturer's protocol.

In this bead-based detection method, the sample is mixed with prefabricated colored, magnetic beads in a 96-well plate. The beads were previously coated with analyte-specific capture antibodies. Each bead color is specific for a single analyte. After preparing wash buffer (WB) and universal assay buffer (UAB) according to the manufacturer’s specifications, antigen standards containing the analytes of interest were prepared and serially fourfold diluted to generate a standard curve for quantification in later measurements. Subsequently, magnetic beads were prepared by thoroughly vortexing the bead solution for 30 s. 50 µl of magnetic bead solution was then added to each well of a 96-well plate. The plate was inserted into a hand-held magnetic plate washer (BioTek® Handheld Magnetic Bead Washer, MerckMillipore, Darmstadt, Germany) and let stand for 2 min for the beads to settle. Afterwards, the magnetic beads were washed by discarding the supernatant fluid with a quick inversion of the plate while inserted in the hand-held plate magnet. Coated magnetic beads remained in the wells and were washed with 150 µl of WB. The process was repeated twice. Next, 25 µl of UAB and either 25 µl of prepared standards or sample were added to each well. After adding standards and samples, the plate was sealed, covered with a black microplate lid and incubated at room temperature (RT) for 30 min on a plate shaker set to 500 rpm. The plate was then transferred to a fridge for additional overnight incubation at 4 °C for optimal binding between analytes and coated beads. On the subsequent day, plates were removed from the fridge and again placed on a plate shaker for 30 min at 500 rpm.

Following the incubation and binding of the analytes to their specific antibodies, the magnetic beads were again washed twice as described above. After that 25 µl of detection antibody mixture containing biotinylated detection antibodies were added to each well, the plate sealed, covered with a black lid and incubated again for 30 min on a plate shaker at 500 rpm at RT. This results in the formation of an analyte-antibody sandwich complex. Following incubation two additional wash steps were performed and 50 µl of streptavidin–phycoerythrin (SAPE), a light-excitable substance, was added, which in turn binds to the biotinylated detection antibodies. Following another 30 min of incubation and two final washing steps, 120 µl of reading buffer was added to each well and again incubated, however only for 5 min. After final incubation the analysis was run on the MAGPIX system.

Since only certain colors of the colored magnetic beads can be analyzed together and some analytes need pre-treatment, specific combinations of analyte sets were combined in the pre-mixed kits. The following four combinations were analyzed:

- 4-Plex (MMP- 2, MMP- 3, MMP- 9, TIMP- 1)
- 5-plex (MMP- 1, MMP- 7, MMP- 8, MMP- 12, MMP- 13)
- 26-Plex (EGF, FGF- 2, G-CSF, GM-CSF, IFN-γ, IL- 1α, IL- 1 β, IL- 1RA, IL- 4, IL- 6, IL- 8 (CXCL8), IL- 9, IL- 10, IL- 13, IL- 17 A, IL- 18, IL- 21, IP- 10 (CXCL10), MCP- 1 (CCL2), MIP- 1α (CCL3), MIP- 1β (CCL4), PDGF-BB, TGF-α, TNF-α, TNF-β, VEGF-A)

Due to naturally high concentrations of the analytes of the 4-plex in biological samples and detection limits, samples had to be diluted 100-fold using the UAB before running the experiments.

During analysis in the Luminex MAGPIX system, beads are aligned in the wells using a magnet and exposed to lasers of varying wavelengths. A red laser (635 nm) is employed to excite the colored beads and identify the color of the analyte to be measured. A green laser (532 nm) is employed to excite the bound streptavidin-PE complexes, whereby the intensity of the streptavidin-PE signal is directly proportional to the concentration of the specific analyte enabling quantification. The measurement is conducted using a charge-coupled device (CCD) camera. A minimum of 50 bead events measured were set as baseline to use quantification results in subsequent analyses. Quantification is conducted via the integrated xPONENT® for MAGPIX software, wherein measurements were compared against a standard curve constructed from the analyte’s standard quantification.

### Statistical analyses

Where appropriate, mean ± standard deviation (SD), median and range or counts (n) and percentages (%) are reported for study cohort characteristics. Total protein quantification is presented as median values with range (min–max) in mg/ml, while specific analyte concentrations were assessed in pg/ml. Samples were measured in technical duplicates (n = 2) for each analyte. The data was analyzed using GraphPad Prism (version 10.2.3; GraphPad Software LLC, Boston, USA). The values of analyte concentrations are presented as median ± interquartile range (IQR). In graphs the scale was adjusted to a log_10_ basis, and results shown as log_10_(pg/ml) for better visualization due to different reference scales for each analyte.

A total of 112 patients was planned to be recruited in the cross-sectional immunomarker observation study. Sample size estimation and calculation was thereby based on planed statistical analyses of binary outcome variables (healing vs. non-healing using for example a two-sided t-test) or categorical variables (wound entities using for example one-way ANOVA). Thereby an average power of at least 0.8, an α-level of 0.05% and a medium to large effect size of 0.6 (Cohen’s d) or 0.4 (Cohen’s f statistic) was assumed. The effect size assumptions are based on previously performed studies in the and preliminary data. This yielded a total sample size of 90 patients for binary outcome variables and 98 for multiple group comparisons. To account for expected heterogeneity within and among the groups and the potential of a non-normal distribution of data, an oversizing of the study group of 15% was employed, leading to a total sample size of 112 patients. Based on these calculations a minimum group size of 8 samples per group was necessary. The program G*Power (version 3.1.9.7, [21]) was used for sample size calculations.

Data was assessed graphically for normal distribution, which was not consistently given, therefore nonparametric statistical test alternatives were employed. For ordinally scaled variables (‘stage’, ‘entity’ and ‘infection’), results were analyzed using the Kruskal–Wallis test with Dunn’s post-hoc test for multiple comparison adjustment. Binary scaled outcome variables (‘status’) were compared using a two-tailed Mann–Whitney U test. An alpha level of 0.05 (5%) was assumed for statistical significance. Heatmaps were created in R (R version 4.3.2 [22]) using the “ComplexHeatmap” package [23]. Mean fluorescence intensity (MFI) scores were log_10_ transformed and z-scored for analyses. The canberra-method for distance with complete linkage was used for hierarchical clustering and n = 3 cluster selected.

In addition to single marker concentrations, ratios of opposing markers based on the example of the MMP- 9/TIMP- 1 ratio [24–26], were explored. The rational hereby is to set markers into relation with each other that represent opposing parts of the regenerative process such as a pro-inflammatory aspect and a pro-regenerative aspect. By doing so, excessive changes in one part of the ratio highlight a disbalance in the process and might be used as predictive monitoring biomarker. Apart from the already proposed MMP- 9/TIMP- 1 ratio, the newly proposed ratios IL- 1 beta/IL- 1RA and CXCL- 8/CXCL- 10 were explored in this study. Area under the curve (AUC) calculations and receiver-operator curve (ROC) generation was performed using GraphPad Prism with simple logistic regression analysis to evaluate predictive ratios. The Youden-Index was used to establish best cut-off values for the predictive ratios and determine sensitivity and specificity.

## Results

A total of 112 samples from patients recruited in the ‘Wound-BIOME’ cohort were analyzed spanning seven different wound entities. Of those, 110 samples were included in the analysis, two were excluded due to measurement errors and partial missing data. **Supplementary Table 1** depicts a breakdown of the cohorts’ variables and **supplementary Table 2** summarizes the raw quantitative data measured for each analyte and outcome parameter.

### Clinical parameters

Table 2 summarizes patient demographics and clinical parameters in total as well as stratified based on wound entities.

**Table 2:** Patient cohort baseline characteristics for complete dataset in total and stratified based on wound entities

Variable	Total (n = 110)	AW (n = 10)	WHD (n = 20)	ALU (n = 15)	VLU (n = 30)	DFU (n = 15)	MIX (n = 8)	PG (n = 12)	*p*-value^#^
Age (years)	68.37 ± 13.23	65.20 ± 8.69	65.95 ± 15.77	73.87 ± 9.93	68.10 ± 14.30	72.73 ± 8.09	73.38 ± 11.60	60.08 ± 15.10	.122
Gender									.746
Male	59 (53.64%)	5 (50.0%)	9 (45.0%)	10 (66.67%)	15 (50.0%)	12 (80.0%)	5 (62.50%)	3 (25.0%)	
Female	51 (46.36%)	5 (50.0%)	11 (55.0%)	5 (33.33%)	15 (50.0%)	3 (20.0%)	3 (37.50%)	9 (75.0%)	
Wound age (in weeks)	97.04 ± 164.52	1.20 ± 0.42	105.70 ± 181.19	95.13 ± 134.63	133.57 ± 217.92	27.00 ± 25.39	165.88 ± 158.19	115.17 ± 154.46	* <.0001
Wound area (in cm^2^)	54.67 ± 115.34	10.40 ± 3.63	26.38 ± 46.85	17.20 ± 13.61	101.55 ± 170.41	19.45 ± 20.53	58.69 ± 60.41	109.69 ± 179.51	*.0.034
Healing status									*.0002
Healing	47 (42.73%)	10 (100.0%)	12 (60.0%)	3 (20.0%)	10 (33.33%)	7 (46.67%)	0 (0.0%)	5 (41.67%)	
Non-Healing	63 (57.27%)	0 (0.0%)	8 (40.0%)	12 (80.0%)	20 (66.67%)	8 (53.33%)	8 (100.0%)	7 (58.33%)	
Regeneration stage									* <.0001
Infection	13 (11.82%)	0 (0.0%)	0 (0.0%)	4 (26.67%)	4 (13.33%)	3 (20.0%)	0 (0.0%)	2 (16.67%)	
Inflammation	41 (37.27%)	0 (0.0%)	3 (15.0%)	6 (40.0%)	14 (46.67%)	6 (40.0%)	7 (87.50%)	5 (41.67%)	
Proliferation	40 (36.36%)	10 (100.0%)	8 (40.0%)	4 (26.67%)	9 (30.0%)	4 (26.67%)	1 (12.50%)	4 (33.33%)	
Epithelization	16 (14.55%)	0 (0.0%)	9 (45.0%)	1 (6.67%)	3 (10.0%)	2 (13.33%)	0 (0.0%)	1 (8.33%)	
Infection status									*.0003
Non-Infected	40 (36.36%)	10 (100.0%)	10 (50.0%)	4 (26.67%)	8 (26.67%)	6 (40.0%)	0 (0.0%)	2 (16.67%)	
Colonization	57 (51.82%)	0 (0.0%)	10 (50.0%)	7 (46.67%)	18 (60.0%)	6 (40.0%)	8 (100.0%)	8 (66.67%)	
Infected	13 (11.82%)	0 (0.0%)	0 (0.0%)	4 (26.67%)	4 (13.33%)	3 (20.0%)	0 (0.0%)	2 (16.67%)	

Categorical variables are presented as fraction of total (%); continuous variables are presented as mean ± standard deviation (SD)

^#^—analysis was performed using Chi-squared test for categorical variables and Kruskal–Wallis test as non-parametric test for continuous variables to compare differences in demographic variables between entities. A significance level of p <.05 was used for all comparisons (*p ≤.05; **p ≤.01; ***p ≤.001; ****p ≤.0001)

Wound entities showed no significant differences regarding the populations age or gender distribution. Mean age of the cohort was 68.37 ± 13.23 and 59 males (53.64%) vs. 51 females (46.36%) were sampled. Entity subgroups were balanced, except for arterial leg ulcers (ALU) and diabetic foot ulcers (DFU) which showed a predominantly male population (10 vs. 5 and 12 vs. 3). Conversely, the pyoderma gangrenosum (PG) group comprised more females than males (3 vs. 9). The mean wound age (time of existence in weeks) was 97.04 weeks with a range of 1 to 1196 weeks (23 years). Acute wounds (as per definition) demonstrated a significantly shorter wound age than chronic entities with a mean of 1.20 ± 0.42 weeks. Interestingly, DFUs also showed a short wound age with 27.00 ± 25.39 weeks. The longest mean duration was observed for arterio-venous/mixed ulcers (MIX) with 165.88 ± 158.19 weeks. Total wound area (assessed in cm^2^ – length x width) showed a mean of 54.67 ± 115.34 cm^2^, whereby acute wounds demonstrated the smallest overall area (10.40 ± 3.63 cm^2^). VLUs and PGs demonstrated the largest wound area with 101.55 ± 170.41 and 109.69 ± 179.51, respectively.

With 47/110 wounds (42.73%) deemed healing and 63/110 (57.27%) non-healing, groups were balanced in terms of healing status. Due to the observational study design, subgroups were unbalanced across different entities. This can also be explained by the fact that acute wounds were all evaluated as healing, as they function as a control group for this study. Most observed wounds were in the inflammatory (37.27%) and proliferative stage (36.36%) while infected (11.82%) and epithelializing wounds (14.55%) were less represented in the overall cohort. Subgroups reflected a similar distribution with the exception of acute wounds, which were all categorized as proliferating (again reflecting the subgroups role as control group). Lastly, most evaluated wounds in the cohort were non-infected (36.36%—40/110) or colonized (51.82%—57/110). Only a small number of wounds were assessed as infected at the time of sampling (11.82%—13/110).

### Total protein quantity

The median total protein concentration in the wound swab samples was 20.14 mg/ml (0.96–94.30). The results were heterogeneous across the entities (Table 3) with 33.35 mg/ml (17.14–43.74) in acute wounds (AW), 8.31 mg/ml (1.19–94.30) in wound healing disorder (WHD), 16.63 mg/ml (5.49–37.64) in arterial ulcer (ALU), 22.76 mg/ml (0.96–85.57) in venous ulcer (VLU), 18.23 mg/ml (4.53–39.45) in diabetic foot ulcer (DFU), 26.67 mg/ml (10.43–56.12) in pyoderma gangrenosum (PG) and 34.28 mg/ml (16.63–63.53) in arterio-venous/mixed ulcer (MIX). There was however no significant difference in protein concentrations between the investigated entities.

**Table 3:** Total protein concentration in wound swab samples

	Median	Min	Max	n
AW	33.35	17.14	43.74	*7*
WHD	8.305	1.190	94.30	*18*
ALU	16.63	5.490	37.64	*13*
VLU	22.76	0.9600	85.57	*25*
DFU	18.23	4.530	39.45	*9*
MIX	34.28	16.63	63.53	*6*
PG	26.67	10.43	56.12	*9*
TOTAL	20.14	0.9600	94.30	*87*

*AW* acute wound, *WHD* wound healing disorder, *ALU* arterial ulcer, *VLU *venous leg ulcer, *DFU* diabetic foot ulcer, *MIX* mixed arterio-venous ulcer, *PG* pyoderma gangrenosum

### Immunomarker distribution pattern

**Fig. 2:**
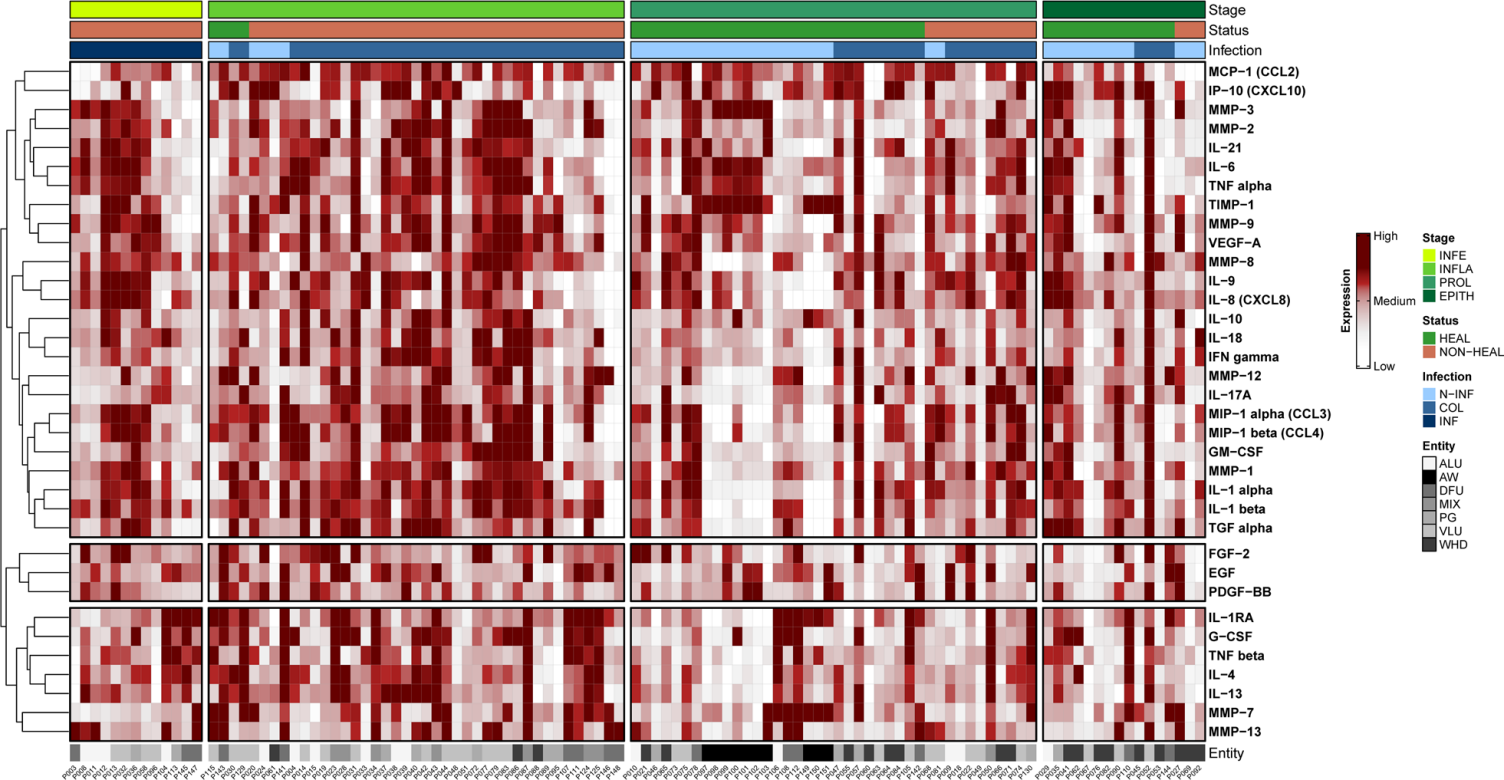
Heatmap of immunomarker measurements in swab samples stratified by clinical outcome parameter. Quantification results of all evaluated immunomarker are shown. Each column represents the swab sample of a patient, corresponding entities are shown in the bottom annotations. Data was hierarchically stratified on the column level based on clinical assessment parameters into healing stage (from left to right), healing status (healing vs. non-healing) and microbial burden. On the row level, hierarchical clustering using the canberra method for distance with complete linkage was performed selecting n = 3 cluster. Lower concentrations of marker are depicted in lighter color, higher expression in darker red. Data is based on mean fluorescence intensity (MFI) which were log_10_-transformed and z-scored. Overall, non-healing wounds in earlier stages and with increased microbial burden demonstrated higher immunomarker expression. (*INFE* infection, *INFLA* inflammation, *PROL* proliferation, *EPITH* epithelization, *N-INF* non-infected, *COL* colonized, *INF* infected, *ALU* arterial leg ulcer, *AW* acute wound, *DFU* diabetic foot ulcer, *MIX* mixed/arterio-venous leg ulcer, *PG* pyoderma gangrenosum, *VLU* venous leg ulcer, *WHD* post-surgical wound healing disorder)

To gain an overview of the expression distribution of immunomarkers a heatmap clustering was performed (Fig. 2). Samples were hierarchically stratified based on the clinical outcome parameters (regenerative stage, healing status and infection status) and clustered on the row level using the canberra distance method with complete linkage. Especially infected and inflamed, non-healing wounds demonstrated a high expression level of inflammatory markers compared to non-infected, healing or acute wounds in the proliferative or epithelization stage. Based on hierarchical clustering, three cluster can be distinguished: Cluster A including cytokines of type 2 immune response (IL- 1RA, G-CSF, TNF beta, IL- 4, IL- 13, MMP- 7, MMP- 13), cluster B containing most growth factors (FGF- 2, EGF, PDGF-BB) and cluster 3 mainly consisting of type 1 and 3 immune response markers and chemokines associated with pro-inflammatory processes. Most acute wounds and wound healing disorders were clustered to the right of the heatmap showing lower expression levels of inflammatory markers (lighter coloring) compared to non-healing or infected wounds, which show an increased expression of pro-inflammatory markers (darker coloring).

### Immunomarker distribution based on wound entity differentiation

**Fig. 3:**
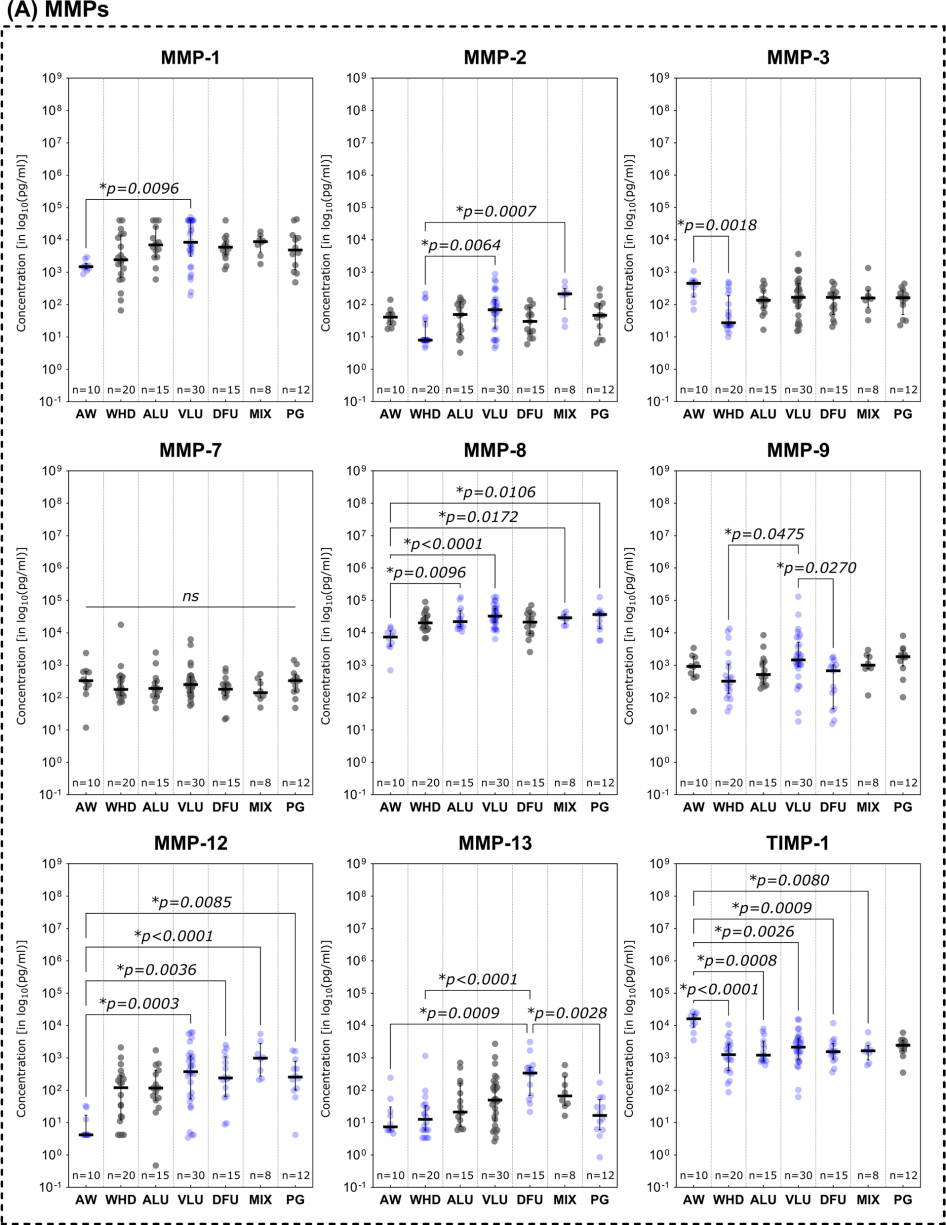

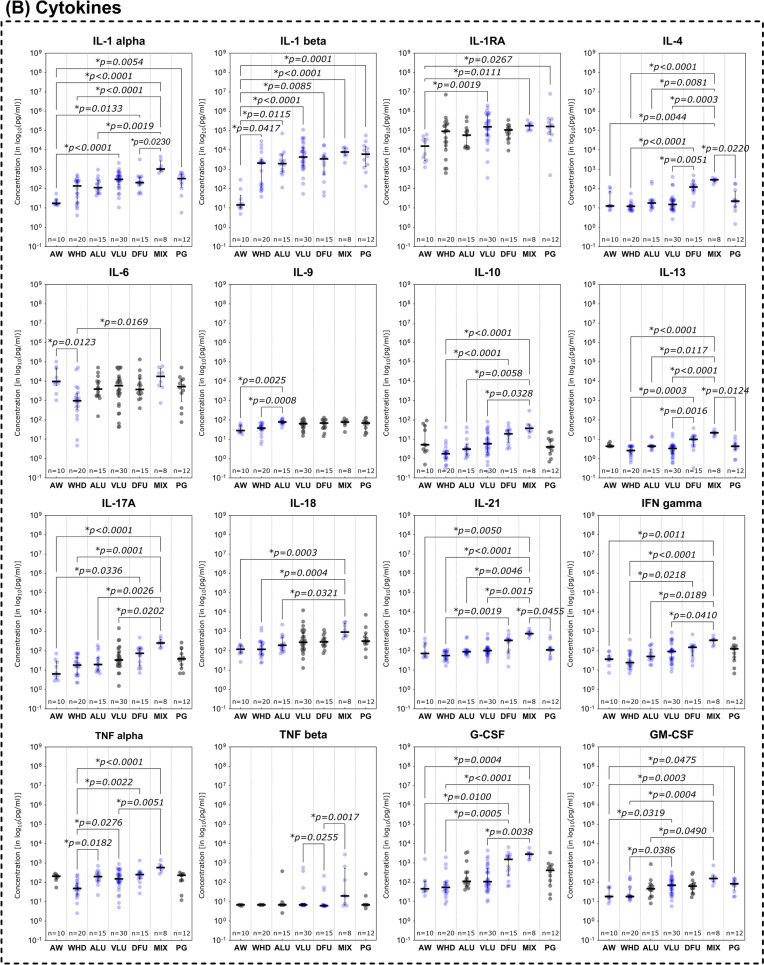

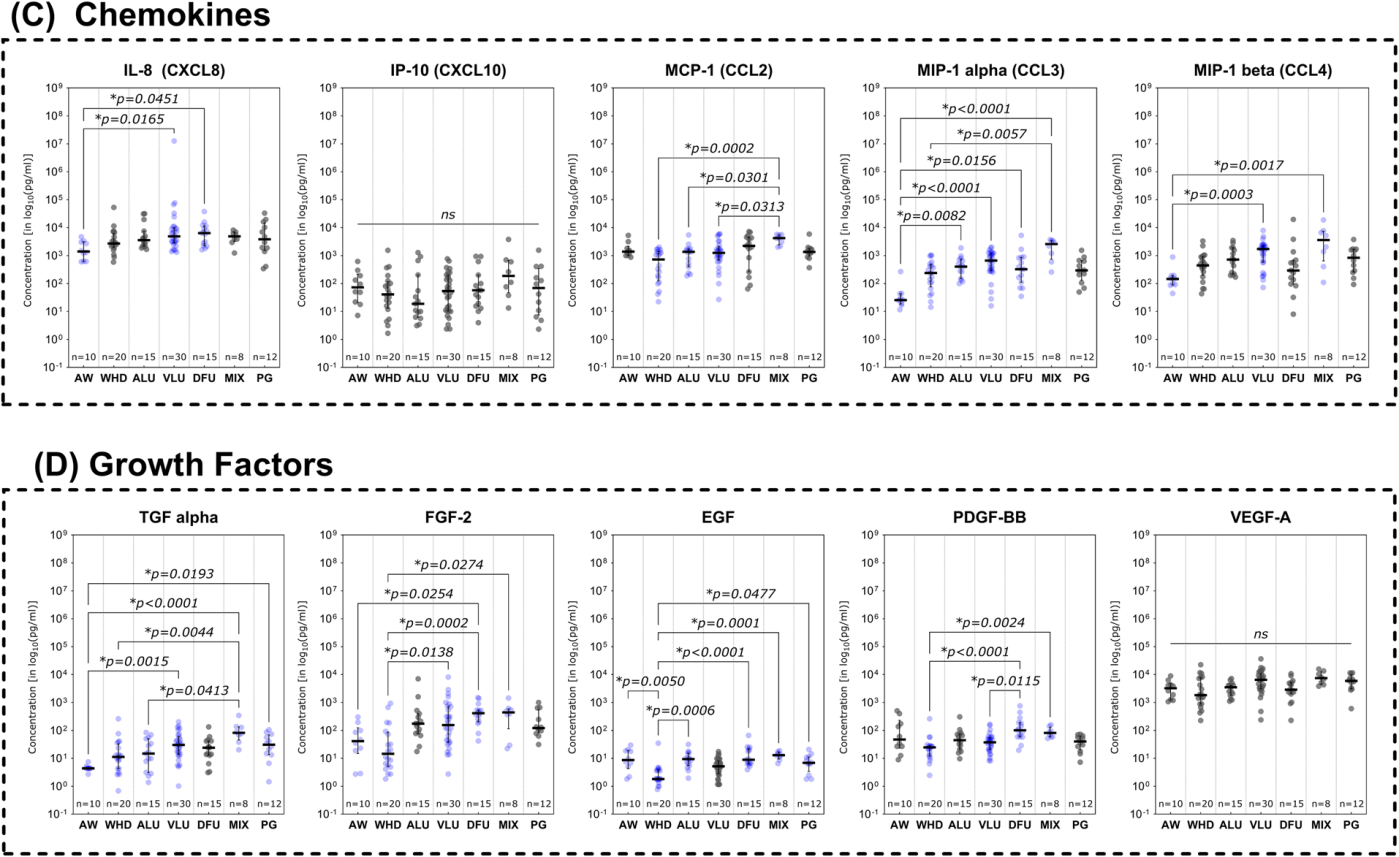
Differences in immunomarker expression between wound entities. Immunomarkers stratified into subcategories are shown: **A** matrix-metalloproteases – MMPs, **B** cytokines, **C** chemokines and **D** growth factors. Values are presented as median ± interquartile range (IQR) and log_10_-transformed for better comparability (concentration in pg/ml). Data was analyzed using the non-parametric Kruskal–Wallis test with Dunn’s post-hoc test for multiple test correction at an α-level of 5% (*p* < 0.05). Groups demonstrating a significant difference were highlighted in blue color as compared to groups showing no significant difference (grey). AW and WHD mostly demonstrated significantly lower concentrations of inflammatory markers and MMPs except for some few (MMP- 3, TIMP- 1, MCP- 1, IL- 6, EGF). Especially MIX demonstrated markedly increased pro-inflammatory marker compared to other entities

Expression and distribution patterns based on entity stratification revealed patterns partially specific for certain subgroups of analytes investigated (Fig. 3). MMPs were especially elevated in diseases with a pathologic venous component (VLU and MIX) or in DFU compared to AW and WHD (Fig. 3A). MMP- 1, MMP- 2, MMP- 8, MMP- 9 and MMP- 12 showed significantly higher levels in VLUs compared to AWs or WHDs (*p* < 0.05). VLUs thereby often displayed the highest overall expression of MMPs amongst entities (MMP- 1, MMP- 8, MMP- 9). MMP- 9 also proved to be significantly elevated in VLUs compared to DFUs (*p* = 0.0270). The MIX group demonstrated significantly higher concentrations of MMP- 2, MMP- 8 and MMP- 12 compared to AWs or WHDs. In DFUs, MMP- 12 levels were significantly higher compared to AWs (*p* = 0.0036), while MMP- 13 was significantly elevated compared to AWs, WHDs and PGs (Fig. 3A). Generally, DFUs showed the highest levels of MMP- 13 compared to all other entities. PGs only showed significantly elevated levels of MMP- 8 and MMP- 12 compared to AWs in this cohort. ALUs showed a significantly higher expression compared to any other entity only for MMP- 8 against AWs (*p* = 0.0096). In contrast, AWs demonstrated significantly elevated levels of TIMP- 1 compared to all other entities except for PGs and significantly higher concentrations of MMP- 3 against WHDs only (p = 0.0018).

In the group of investigated cytokines (Fig. 3B), extracting an overall uniform picture regarding distribution pattern is more difficult. However, in most cases, a higher concentration of pro-inflammatory cytokines can be observed in chronic wound entities compared to AWs. In some cases, the same is true against WHDs. This is especially notable for MIX, as this subgroup demonstrates the highest expression levels for most inflammatory markers (IL- 1 alpha, IL- 4, IL- 17 A, IL- 18, IL- 21, G-CSF, GM-CSF, IFN gamma and TGF alpha) compared to AWs. Especially analytes of the Interleukin- 1 family (IL- 1 family; IL- 1 alpha, IL- 1 beta, IL- 1RA and IL- 18) showed increased levels in chronic wound entities compared to AWs. PGs however only showed significantly elevated levels of GM-CSF (*p* = 0.0475) and TGF alpha (*p* = 0.0193) compared to AWs. Interestingly, AWs demonstrated higher levels of IL- 6 and IL- 10 compared to some chronic wound entities. However, these differences in IL- 6 levels were only statistically significant against WHDs (*p* = 0.0123).

Significantly elevated levels of chemokines were observed for MIX compared to AW or WHD (Fig. 3C). For CCL2, CCL3 and CCL4, MIXs showed the highest expression levels, for CCL2 even significantly higher than other chronic wound entities (vs. ALU and VLU). CCL2 itself showed a comparably high expression in AWs compared to other measured chemokine levels. VLUs and DFUs showed high levels of chemokine expression compared to AWs with significantly higher expression levels for CCL3, CCL4 (only VLU) and CXCL8. For CCL3, all chronic wound entities demonstrated markedly higher expression levels compared to AWs. However, for PG these levels were not statistically significant.

A different pattern was observed for growth factors (Fig. 3D). AWs showed higher expression levels compared to chronic wound entities for EGF and PDGF-BB. Thereby, EGF was especially depleted in WHDs compared to AWs as well as other chronic wound entities (ALU, DFU, MIX, PG). PDGF-BB demonstrated a similar pattern with WHDs demonstrating the overall lowest expression levels with significantly reduced levels compared to DFUs and MIXs. For FGF- 2 however, AWs and WHDs both demonstrated markedly lower concentrations than chronic wound entities. VLUs, DFUs and MIXs thereby showed significantly higher levels of FGF- 2 compared to WHDs. In DFUs FGF- 2 was also significantly increased compared to AWs (*p* = 0.0254).

### Immunomarkers in healing vs. non-healing wounds

**Fig. 4:**
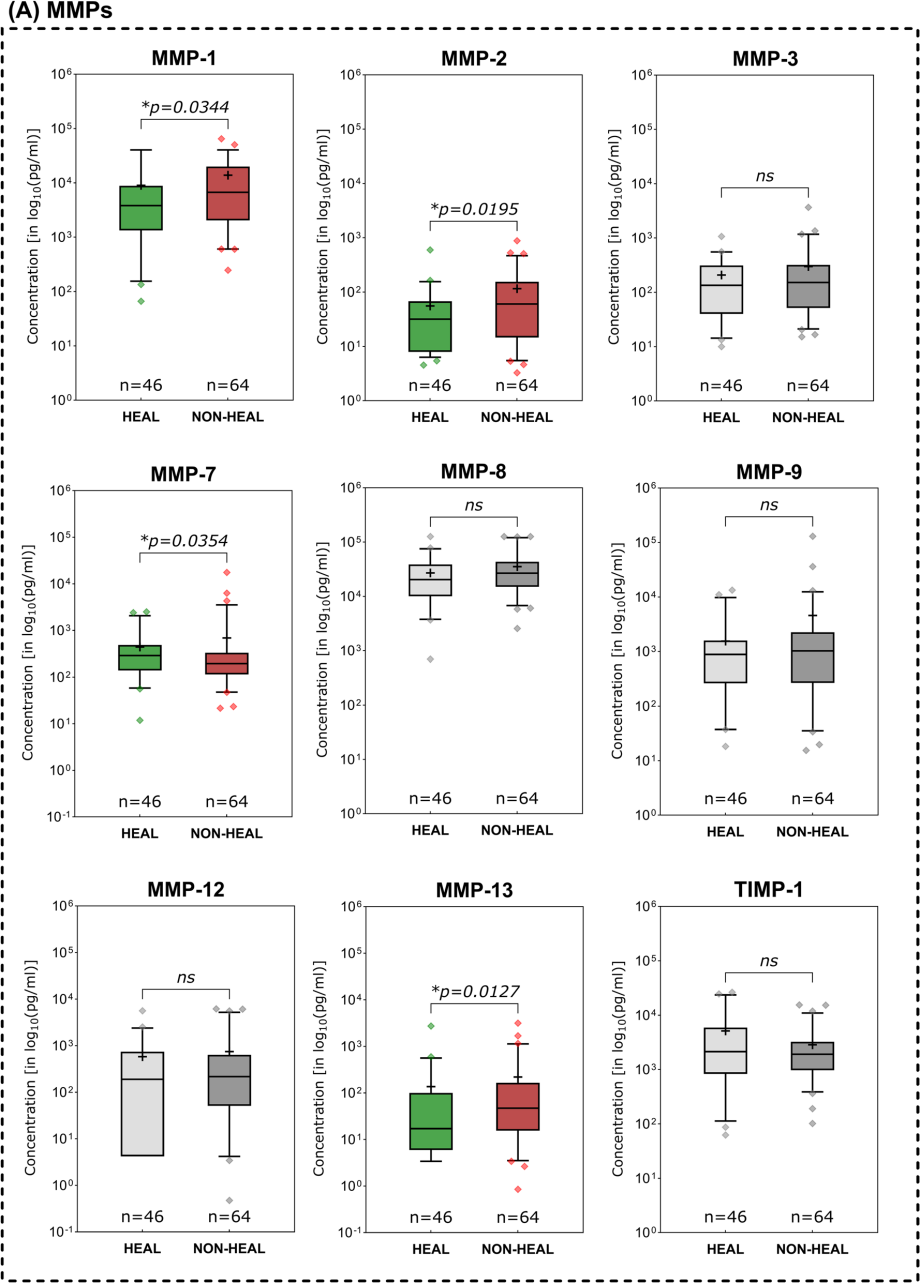

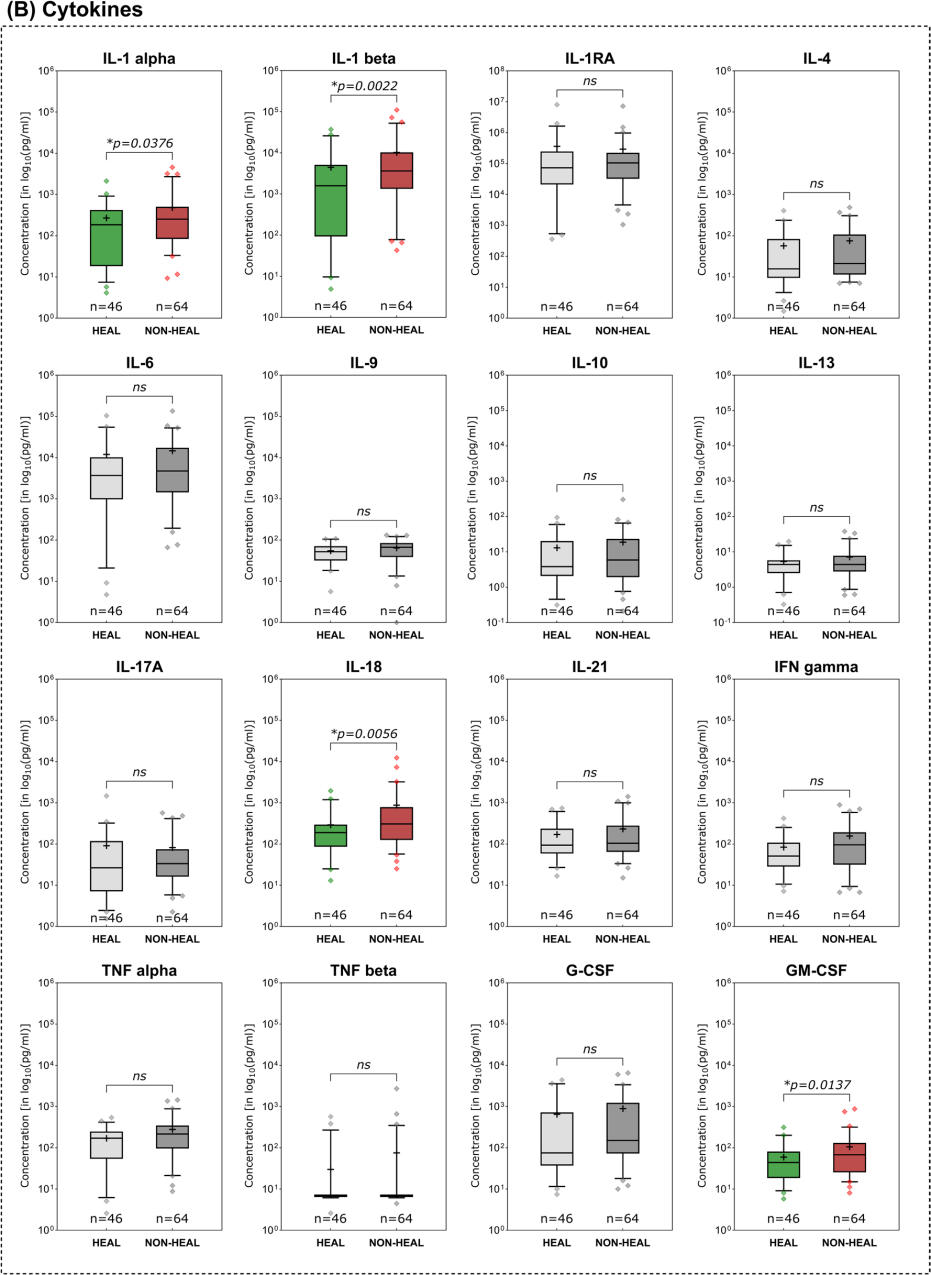

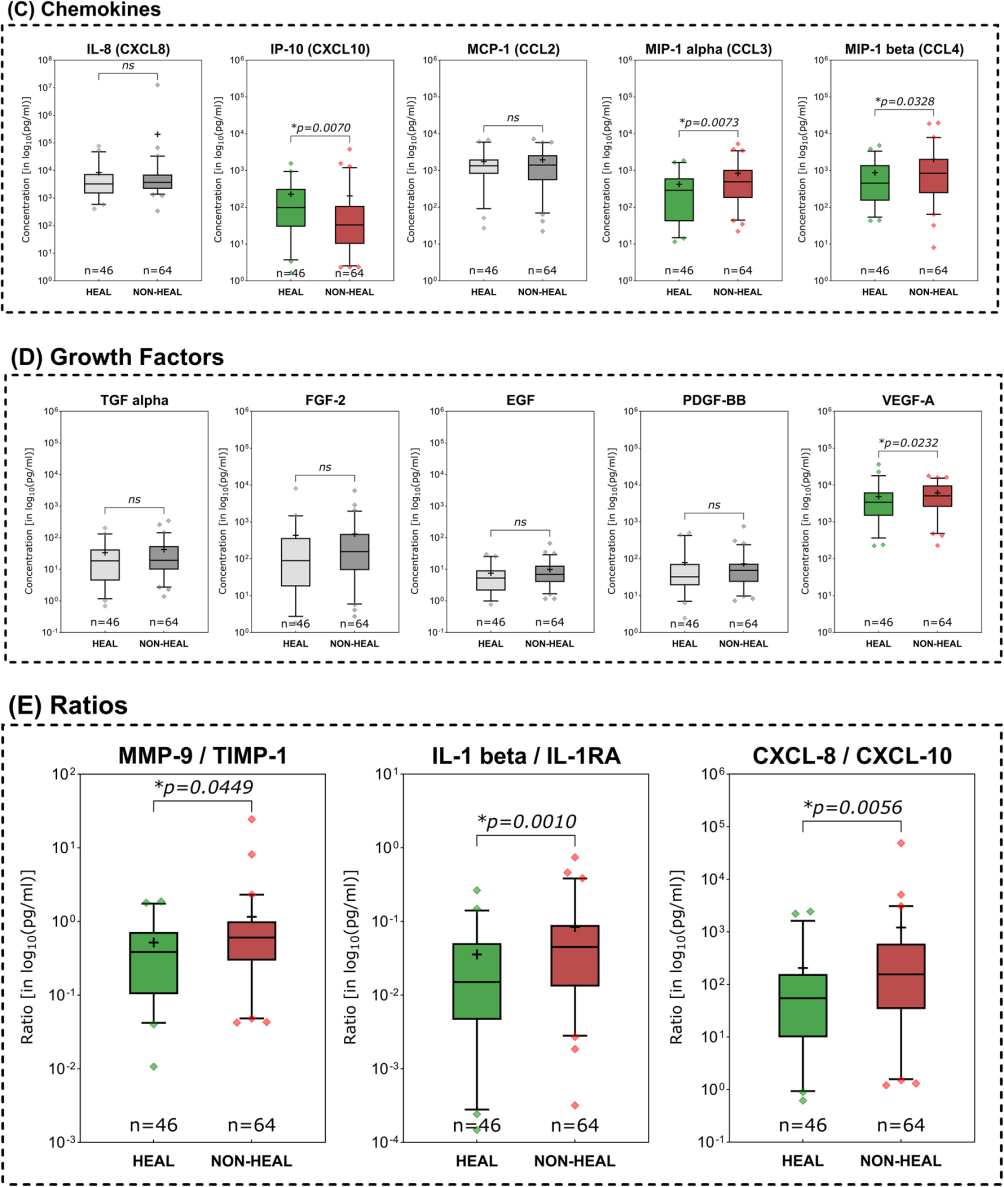
Differences in immunomarker expression between healing and non-healing wounds. Immunomarker expression levels between healing and non-healing wounds are shown: **A** matrix-metalloproteases – MMPs, **B** cytokines, **C** chemokines, **D** growth factors and **E** immunomarker ratios. Values are presented as boxplots with vertical line as median and whiskers representing 5 th to 95 th percentile. ‘ + ’ sign represents the mean; outliers are shown as diamonds. For better comparability due to varying scales of individual analytes, a log_10_-transformation was employed on the y-axis (log_10_(pg/ml)). Raw, untransformed data are presented in supplementary Table 2. Data was analyzed using the non-parametric Mann–Whitney U test at an α-level of 5% (*p* < 0.05). Markers demonstrating a significant difference were highlighted in color as compared to groups showing no significant difference (greyscale). Mostly pro-inflammatory markers and proteases are significantly elevated in non-healing wounds (NON-HEAL) compared to healing wounds (HEAL). Only MMP- 7 and IP- 10 (CXCL- 10) demonstrated significantly higher concentrations in healing wounds than non-healing wounds

Non-healing compared to healing wounds demonstrated several pro-inflammatory immunomarkers to be elevated (Fig. 4). Among investigated MMPs (Fig. 4A), MMP- 1 (*p* = 0.0344), MMP- 2 (*p* = 0.0195) and MMP- 13 (*p* = 0.127) showed significantly higher expression levels in non-healing compared to healing wounds. Only MMP- 7 levels were significantly lower in non-healing wounds (*p* = 0.0354). In terms of cytokines (Fig. 4B), members of the Interleukin- 1 family were significantly elevated in non-healing wounds (IL- 1 alpha, IL- 1 beta and IL- 18). Also, the pro-inflammatory cytokine GM-CSF, a stimulator of granulocyte and macrophage differentiation and proliferation, was observed to be significantly increased in non-healing wounds (*p* = 0.0137). Among chemokines, CCL3 (MIP- 1 alpha) and CCL4 (MIP- 1 beta), both relevant in the immune response to infection and inflammation, were significantly higher expressed in non-healing wounds (*p* = 0.0073 and *p* = 0.0328 respectively, Fig. 4C). Conversely, CXCL10 (IP- 10) was observed to be significantly elevated in healing wounds (*p* = 0.0070). As for growth factors (Fig. 4D), only VEGF-A demonstrated significantly differing levels between healing and non-healing wounds with a higher expression in non-healing wounds (*p* = 0.0232). The evaluated immunomarker ratios MMP- 9/TIMP- 1, IL- 1 beta/IL- 1RA and CXCL- 8/CXCL- 10 all proved to be significantly elevated in non-healing wounds compared to healing wounds (Fig. 4E).

### Immunomarkers in wounds correlated to the regenerative stage

**Fig. 5:**
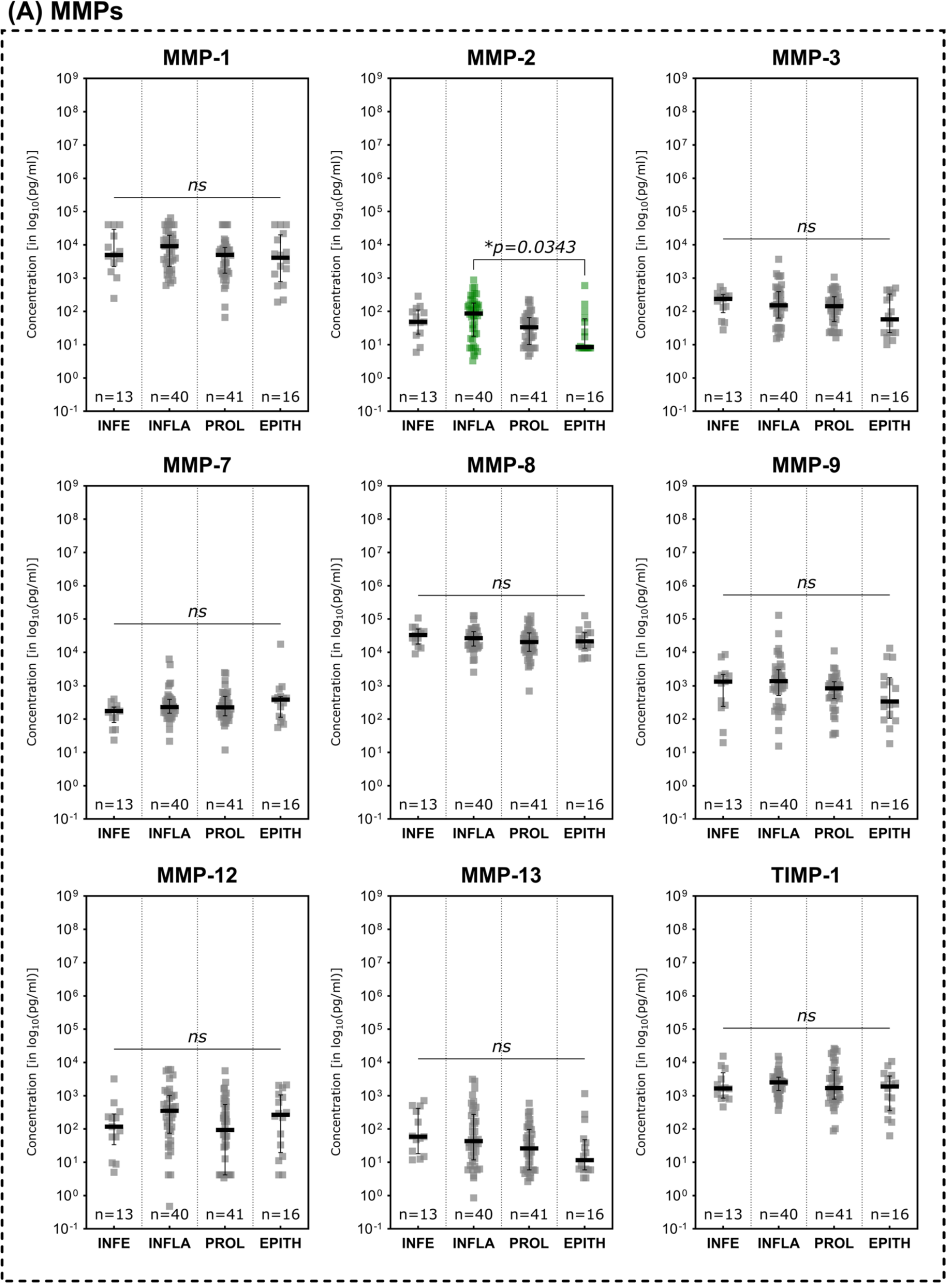

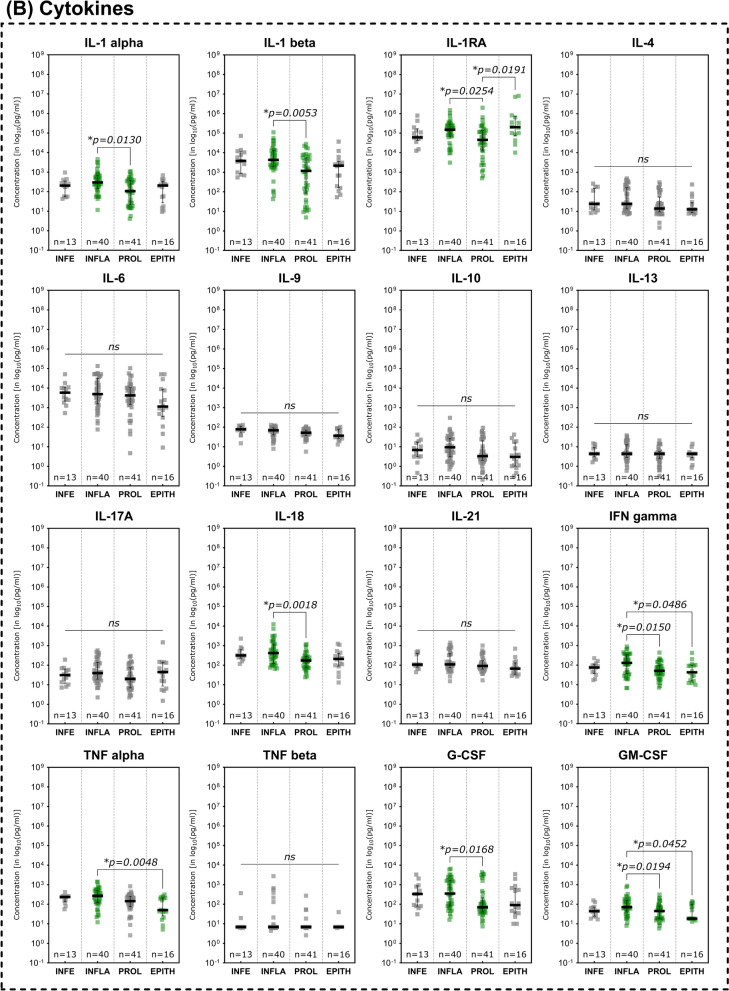

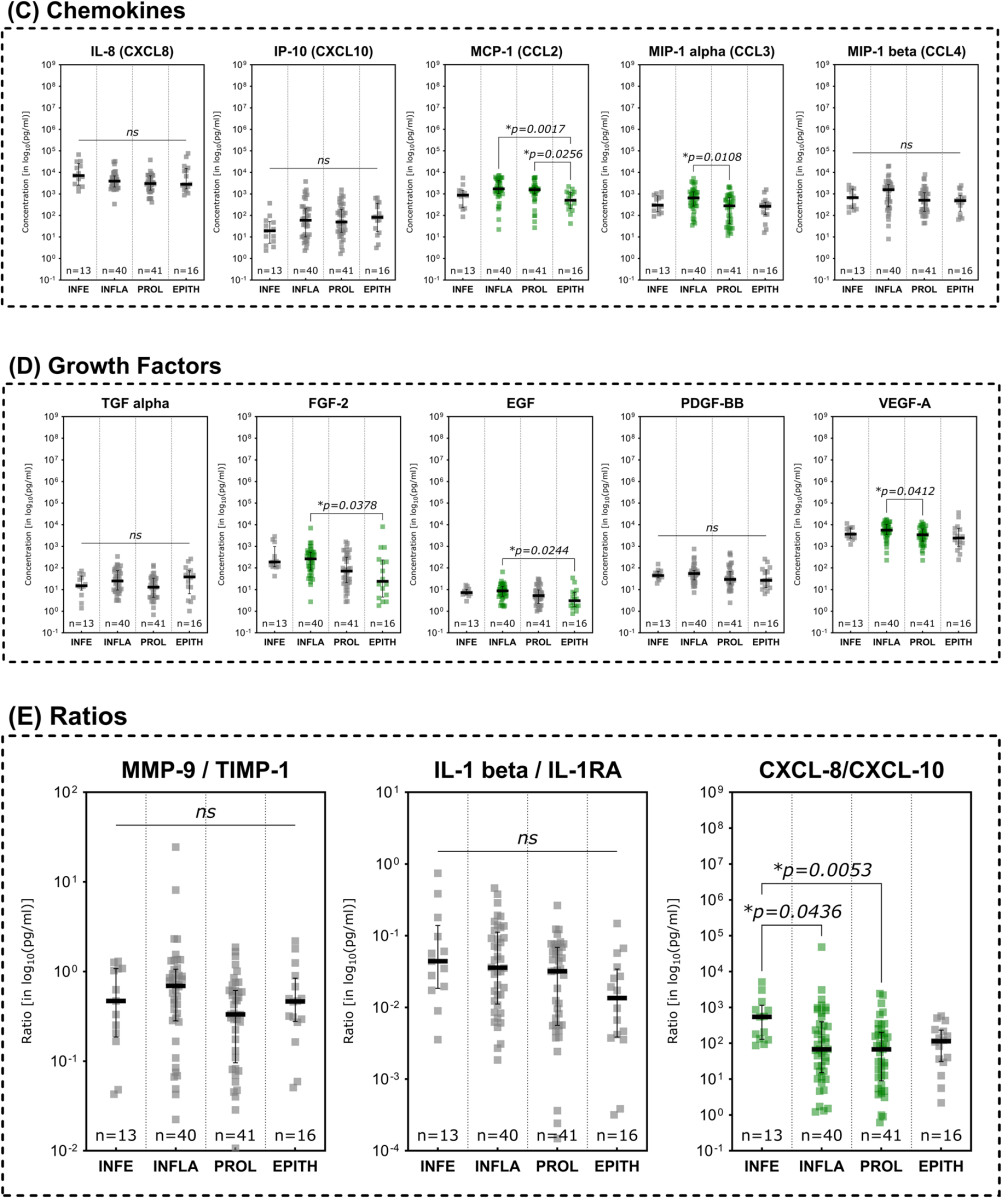
Differences in immunomarker expression across regenerative stages. Immunomarkers stratified into subcategories are shown: **A** matrix-metalloproteases – MMPs, **B** cytokines, **C** chemokines, **D** growth factors and **E** immunomarker ratios. Values are presented as median ± interquartile range (IQR) and log_10_-transformed for better comparability (concentration in pg/ml). Data was analyzed using the non-parametric Kruskal–Wallis test with Dunn’s post-hoc test for multiple test correction at an α-level of 5% (*p* < 0.05). Stages demonstrating a significant difference were highlighted in green color as compared to groups showing no significant difference (grey). Wounds in the inflammatory stage predominantly demonstrated elevated concentrations compared to later stages such as proliferative and epithelization stage, indicating a pronounced pro-inflammatory stage as to be expected of wounds clinically categorized into this stage (*INFE* infection, *INFLA* inflammation, *PROL* proliferation, *EPITH* epithelization)

Investigations of immunomarker across the regenerative stages of the healing process showed an overall reduction of pro-inflammatory marker during the advancing tissue repair process (Fig. 5). While several MMPs demonstrated a continuous decrease over the phases of wound healing, only MMP- 2 showed a significant difference between stages the inflammatory and epithelization stage (Fig. 5A; *p* = 0.0343).

In terms of cytokines (Fig. 5B), again the IL- 1 family was predominantly elevated in wounds in the inflammatory stage. IL- 1 alpha, IL- 1 beta and IL- 18 were significantly higher expressed during the inflammatory stage (*p* = 0.0130, *p* = 0.0053 and *p* = 0.0018 respectively) compared to the proliferative stage. IL- 1RA as natural inhibitor if the IL- 1 induced pro-inflammatory effect on the contrary was significantly higher concentrated in the epithelization stage as compared to the proliferative stage (*p* = 0.0191) and higher concentrated compared to the inflammatory stage (however not statistically significant, *p* > 0.9999). In the inflammatory stage IL- 1RA is also significantly higher expressed than in the proliferative stage (*p* = 0.0254), presumably as part of the holistic immune/inflammation response. Further pro-inflammatory cytokines were also observed to be significantly elevated in the inflammatory stage compared to the proliferative stage (IFN gamma, G-CSF and GM-CSF) or the epithelization stage (IFN gamma, TNF alpha and GM-CSF). Some cytokines thereby demonstrated a continuous decrease in concentration from the inflammatory stage to the epithelization stage (IFN gamma, TNF alpha, GM-CSF).

Chemokines and growth factors showed the same pattern of decreasing concentrations towards later reparative stages (Fig. 5C and 5D). CCL2 (MCP- 1) concentrations were significantly reduced in wounds in the epithelization stage compared to the inflammatory or proliferative stage (*p* = 0.0017 and *p* = 0.0256 respectively). CCL3 (MIP- 1 alpha), concentrations were also significantly higher in the inflammatory stage as compared to the proliferative stage (*p* = 0.0108). EGF and FGF- 2 demonstrated significantly lower concentrations in the epithelization stage as compared to the inflammatory stage (*p* = 0.0244 and *p* = 0.0378 respectively), while VEGF-A showed a significantly reduced expression in the proliferative stage compared to the inflammatory stage (*p* = 0.0412).

While the MMP- 9/TIMP- 1 and IL1 beta/IL- 1RA ratios did not demonstrate significant differences between the stages of the regenerative process, a decreasing pattern could be observed for the IL- 1 beta/IL- 1 RA ratio (Fig. 5E). The CXCL- 8/CXCL- 10 ratio however showed the highest concentration in infected wounds, significantly higher compared to wounds in the inflammatory as well as the proliferative stage (*p* = 0.0436 and *p* = 0.0053 respectively).

### Immunomarkers in infected and non-infected wounds

**Fig. 6:**
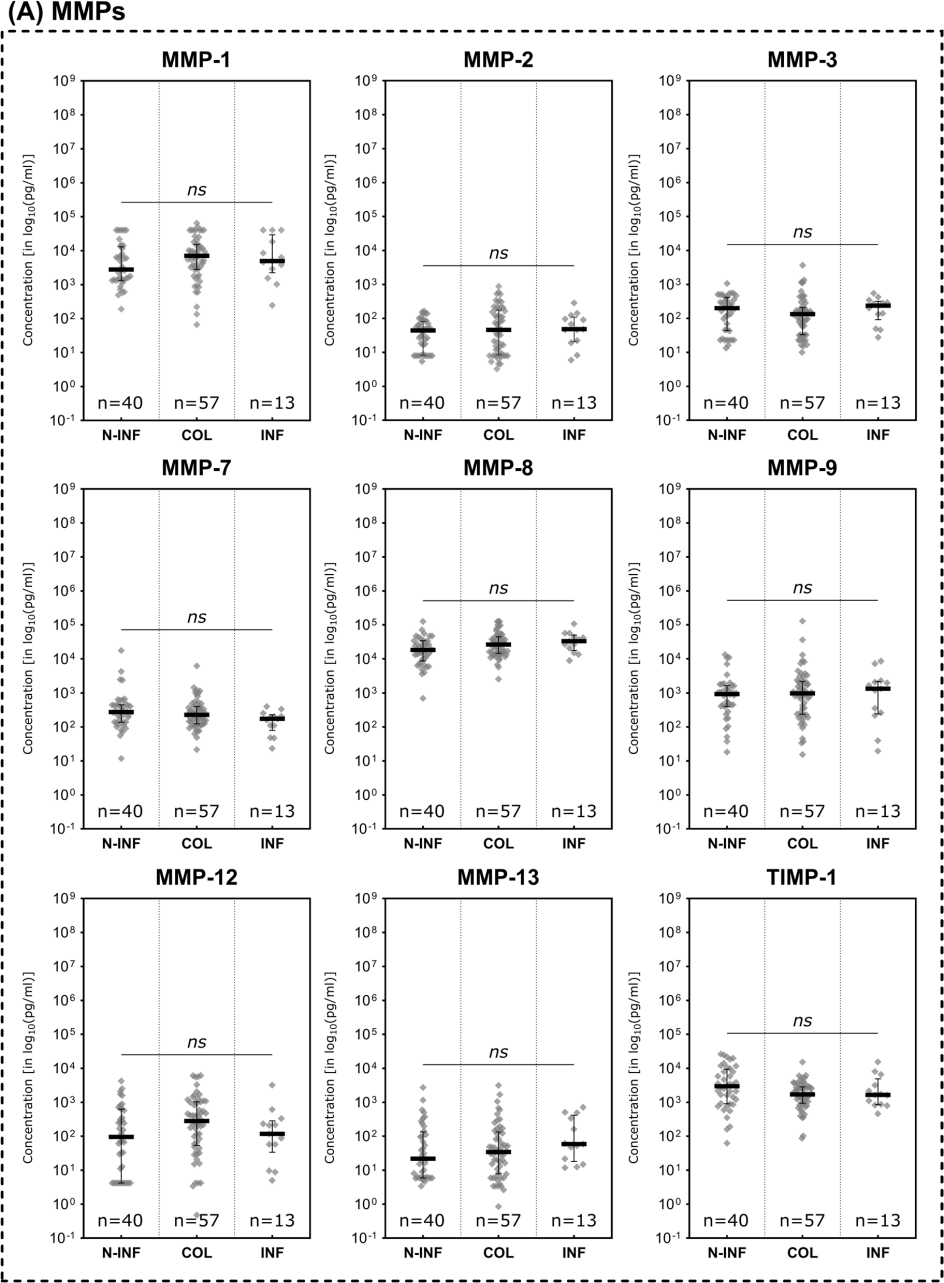

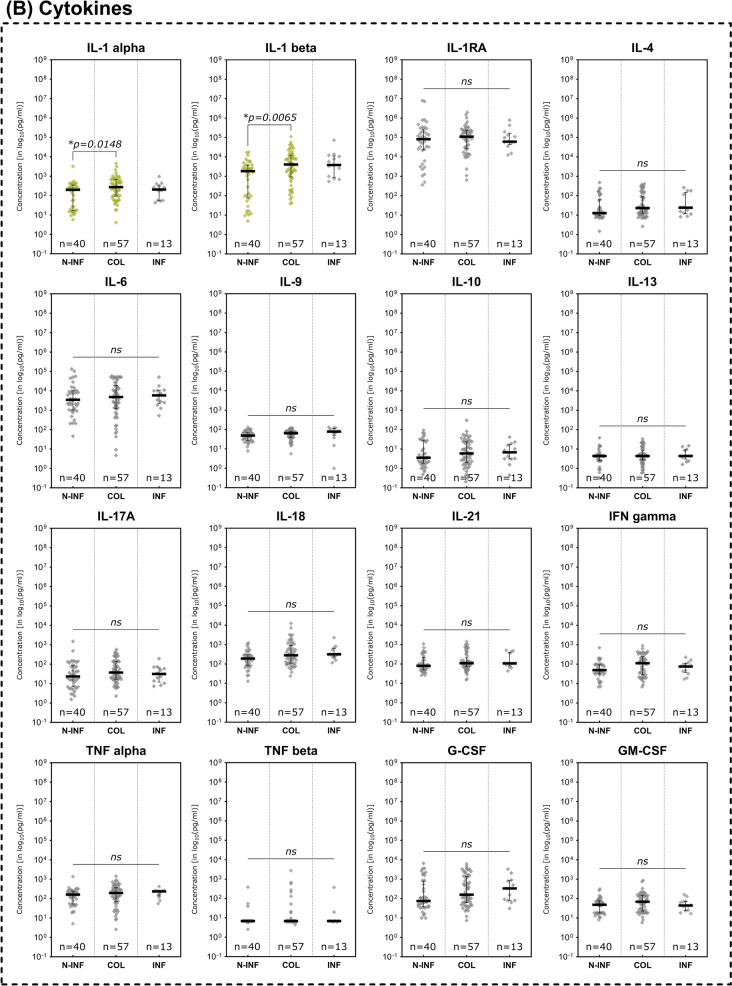

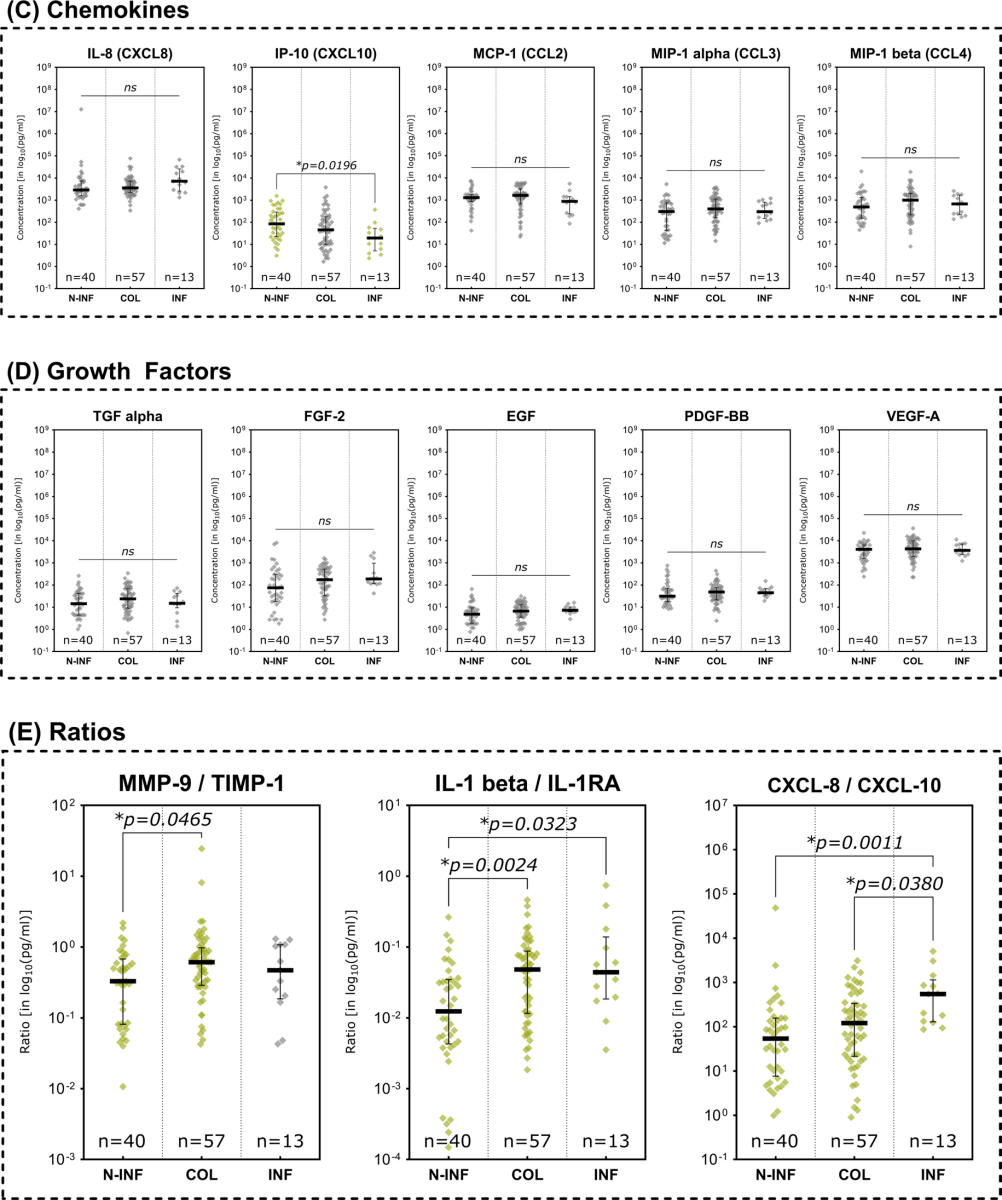
Differences in immunomarker expression between non-infected, colonized and infected wounds. Immunomarkers stratified into subcategories are shown: **A** matrix-metalloproteases – MMPs, **B** cytokines, **C** chemokines, **D** growth factors and **E** immunomarker ratios. Values are presented as median ± interquartile range (IQR) and log_10_-transformed for better comparability (concentration in pg/ml). Data was analyzed using the non-parametric Kruskal–Wallis test with Dunn’s post-hoc test for multiple test correction at an α-level of 5% (*p* < 0.05). Groups demonstrating a significant difference were highlighted in yellow color as compared to groups showing no significant difference (grey). The inflammatory cytokines IL- 1 alpha and IL- 1 beta were significantly lower expressed in non-infected wounds while CXCL10 was significantly higher expressed in non-infected compared to infected wounds (*N-INF* non-infected, *COL* colonized, *INF* infected)

Only three assessed analytes demonstrated statistically significant differences between clinically non-infected, colonized or infected wounds (Fig. 6). MMPs showed no significant differences between subgroups (Fig. 6A). The pro-inflammatory cytokines IL- 1 alpha and IL- 1 beta on the other hand demonstrated significantly higher concentrations in colonized versus non-infected wounds (*p* = 0.0148 and *p* = 0.0065 respectively; Fig. 6B). For both markers, concentrations were also elevated in infected wounds, however the difference towards non-infected wounds was not statistically significant. The chemokine CXCL10 (IP- 10) on the contrary was significantly elevated in non-infected wounds compared to infected wounds (*p* = 0.0196; Fig. 6C). The marker demonstrated a continuous elevation over the different stages of microbial burden from infected to colonized to non-infected. Growth factors showed no significant differences between the compared infectious states (Fig. 6D). The evaluated marker ratios however all demonstrated significantly elevated levels in colonized or infected wounds (Fig. 6E). MMP- 9/TIMP- 1 levels were significantly higher in colonized compared to non-infected wounds (*p* = 0.0465) and the IL- 1 beta/IL- 1RA ratio was significantly elevated in both colonized and infected wounds compared to non-infected wounds (*p* = 0.0024 and *p* = 0.0323 respectively). However, it could not demonstrate a significant difference between colonized and actually infected wounds. The CXCL- 8/CXCL- 10 ratio in contrast demonstrated significantly higher levels in infected wounds as compared to non-infected as well as colonized wounds (*p* = 0.0011 and *p* = 0.0380 respectively).

### Predictive immunomarker ratios

**Fig. 7:**
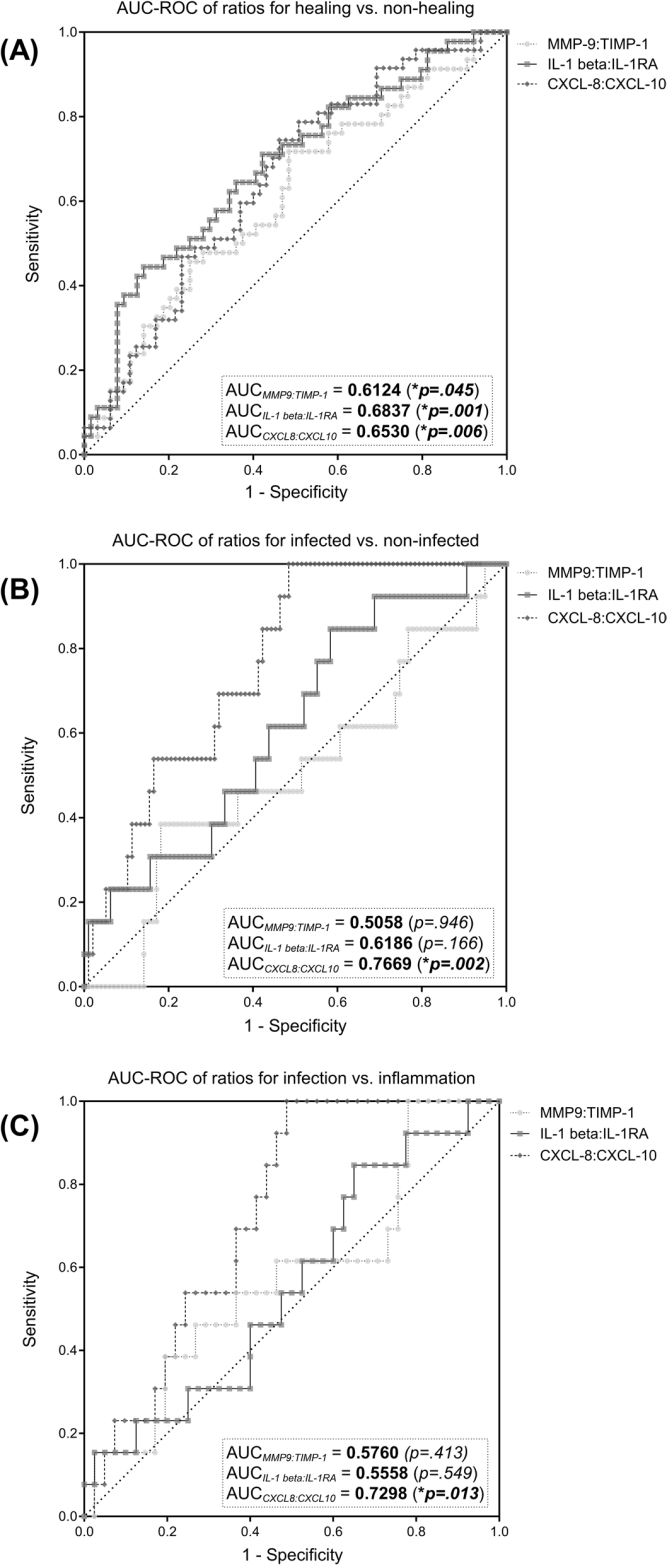
Discriminative capabilities of potential diagnostic marker ratios. Immunomarker ratio MMP- 9/TIMP- 1, IL- 1 beta/IL- 1RA and CXCL- 8/CXCL- 10 for healing vs. non-healing wounds (**a**), infected vs. non-infected wounds (**b**) and infection vs. inflammation (**c**), with potential to function as diagnostic tools were investigated regarding their differential expression. Receiver-operator curves (ROC) were constructed and area under the curve (AUC) calculated to evaluate the discriminative abilities of the ratios. Differences in ratio levels between healing (HEAL) and non-healing (NON-HEAL) wounds **a** are depicted as boxplots with whiskers representing 5 th to 95 th percentile. ‘ + ’ sign represents the mean; outliers are shown as diamonds. Data was analyzed using the non-parametric Mann–Whitney U test at an α-level of 5% (*p* < 0.05). Ratio levels of infected (INF) and non-infected (N-INF) wounds **b** as well as stages (**b**; *INFE* infection, *INFLA* inflammation, *PROL* proliferation, EPITH – epithelization), scatter plots were used with the vertical bar representing the median ± interquartile range (IQR). Data was analyzed using the non-parametric Kruskal–Wallis test with Dunn’s post-hoc test for multiple test correction at an α-level of 5% (*p* < 0.05). For the discrimination between healing and non-healing wounds the IL- 1 beta/IL- 1RA ratio demonstrated the best results (AUC = 0.6837, *p* = 0.001), for infected vs. non-infected wounds and differentiation of infection from inflammation, the CXCL- 8/CXCL- 10 ratio demonstrated the best discriminating abilities with and AUC of 0.7669 (*p* = 0.002) and 0.7298 (*p* = 0.013), respectively

The predictive value of certain immunomarker ratios regarding wound healing status, presence of infection and discrimination between inflammation and actual infection was explored. This approach is inspired by the MMP- 9/TIMP- 1 ratio investigated in previous studies [24–26]. The ratio aims to indicate an excessive proteolytic environment based on increased MMP- 9 levels in relation to depleted levels of its counterpart TIMP- 1. The ratios of IL- 1 beta/IL- 1RA and CXCL- 8/CXCL- 10 were explored based on the same rational, as they represent counterparts in inflammation regulation or accentuate a certain phase of the regenerative process. Figure 7 visualizes the ROC of the investigated ratios for the evaluated comparisons and Table 4 provides a summary of best cut-off values based on the Youden-Index along with corresponding AUC, sensitivity and specificity values.

**Table 4:** Overview for optimal cut-off values for the ratios based on Youden-Index and corresponding AUC, sensitivity and specificity values

		Ratio Cut-Off	AUC	Sensitivity *(in %)*	Specificity *(in %)*	Youden-index
HEAL vs. NON-HEAL	MMP- 9/TIMP- 1	> 0.594	0.6124	51.56	71.74	0.23
	IL- 1 beta/IL- 1RA	> 0.008	0.6837	85.94	44.44	0.30
	CXCL- 8/CXCL- 10	> 139.8	0.6530	53.85	74.47	0.28
INFE vs NON-INFE	MMP- 9/TIMP- 1	< 1.017	0.5058	81.82	38.46	0.20
	IL- 1 beta/IL- 1RA	< 0.017	0.6186	41.67	84.62	0.26
	CXCL- 8/CXCL- 10	< 86.36	0.7669	51.55	100.00	0.52
INFE vs. INFLA	MMP- 9/TIMP- 1	< 0.336	0.5760	46.15	73.17	0.19
	IL- 1 beta/IL- 1RA	> 0.017	0.5558	84.62	35.00	0.20
	CXCL- 8/CXCL- 10	> 77.58	0.7298	100.00	51.22	0.51

*AUC* area under the curve, *HEAL* healing wound, *NON-HEAL* non-healing wound, *INFE* infected wound, *NON-INFE* non-infected wound, *INFLA* inflamed wound

The previously described MMP- 9/TIMP- 1 ratio as a marker of imbalance in the proteolytic environment of a wound only demonstrated a moderate suitability to distinguish between healing and non-healing wounds (AUC = 0.6124, 95%-CI[0.5055; 0.7193]; Fig. 7A). Regarding the discrimination of infected from non-infected wounds (AUC = 0.5058, 95%-CI[0.3321; 0.6796]; Fig. 7B) and infected from inflamed wounds (AUC = 0.5760, 95%-CI[0.3942; 0.7578]; Fig. 7C) the ratio performed even worse.

Of the evaluated ratios, the IL- 1beta/IL- 1RA ratio demonstrated the best discriminative abilities to distinguish healing from non-healing wounds (Fig. 7A) with an AUC of 0.6837 (95%-CI[0.5816; 0.7857]). In terms of infection detection, it performed moderately with an AUC of 0.6186 (95%-CI[0.4627; 0.7745]; Fig. 7B), however showed a poor discrimination between infected and inflamed wounds (AUC = 0.5558; Fig. 7C).

Lastly, the CXCL- 8/CXCL- 10 ratio demonstrated a moderate discrimination between healing and non-healing wounds (AUC = 0.6530, 95%-CI[0.5515; 0.7545]) and therefore ranks second behind the IL- 1 beta/IL- 1RA ratio regarding healing discrimination (Fig. 7A). However, the ratio demonstrated the best suitability for distinguishing infected from non-infected wounds (AUC = 0.7669, 95%-CI[0.6579; 0.8758]; Fig. 7B). Additionally, the CXCL- 8/CXCL- 10 ratio also demonstrated a comparably good discriminative ability in terms of distinguishing between an infected or an inflamed stage (AUC = 0.7298, 95%-CI[0.5963; 0.8634]; Fig. 7C).

## Discussion

Wound repair is a carefully orchestrated process, both temporally and spatially. Numerous players are involved on a cellular and humoral level including immune and tissue cells, cytokines, chemokines, growth factors and components of the ECM (fibronectin, collagen, glycosaminoglycans) [5]. Fine-tuned intercellular crosstalk, cell recruitment and signal transduction aim to restore lost tissue integrity at the site of wounding. In the last couple of decades several studies on each individual aspect of the complex system their interactions have advanced our understanding of this intricate network [27–30]. However, some aspects of the impaired relationships of immunomarker signaling in the context of delayed wound healing remain elusive. Differences in marker expression patterns regarding regenerative stages of the healing process, progression or stagnation of healing and signatures of infection are still not fully understood. This hinders proper diagnosis, intervention allocation and therapeutic monitoring. The improvement of our knowledge regarding immune signatures and the identification of potential steering parameters for individualized therapy is therefore essential [7, 11, 12]. Earlier work targeted specific analytes such as cytokine and growth factor levels [29, 31] or MMP patterns [28] to identify biomarkers in the context of chronic wound healing. Many studies however fall short of examining a holistic picture of the multitude of potential markers, their interconnectedness and association with clinical states. Previous studies were predominantly performed *in-vitro* or in animal models, not in a translational approach using human biomaterial. In those rare cases that human material was examined, invasive biopsies or complex sampling techniques for wound exudate collection were employed. Additionally, only a limited number of recurring analytes and/or entities were included in these studies. Relevant clinical outcome parameters to correlate marker patterns with clinical aspects were rarely collected. Therefore, our study aimed to provide a comprehensive observation of multiple cytokines, chemokines, growth factors and MMPs in various chronic wound entities and clinical stages of the healing process.

Distinctive differences in expression patterns were be observed for various immunomarkers based on entity attribution, regenerative stage or healing tendencies. Marker patterns for example reflected previously reported elevated proteolytic protease levels in certain wound entities. Elevated levels of MMPs in wound biopsies and exudate of VLUs [32–35] as well as DFUs [34–36] have been previously reported as signs of dysregulated ECM remodeling. The level of produced and released MMPs in relation to their counterparts, tissue-inhibitors of matrix-metalloproteases (TIMP), has been described as a driver for delayed tissue regeneration in chronic wounds [17, 37–39]. Specifically, MMP- 1 [32, 35], − 2 [32, 33, 39], − 8 [32, 40], − 9 [26, 32, 39], − 12 [41] and − 13 [14, 42] were demonstrated to be overexpressed in chronic wound models. This phenomenon resulted in the perpetuation of a pro-inflammatory, self-destructive state. Elevated levels of proteases in general [34, 35] and specifically MMP- 13 [14] and the ratio of MMP- 9 to TIMP- 1 [24–26, 43] were therefore proposed as potential markers for delayed wound healing. In line with previous reports [44], our results (Fig. 3A) confirm elevated levels of MMPs in a variety of chronic wound entities compared to AWs and WHDs. These observations underscore the concept of a highly activated proteolytic system in chronically damaged tissue. To a certain extent and at the correct time, elevated levels of proteases are necessary to clear destroyed tissue and cellular debris, facilitate pathogen defense and orchestrate proper tissue restoration and remodeling [28]. Therefore, MMPs play a vital role in fibrogenesis, angiogenesis and immune reaction. However, a prolonged and excessive proteolytic activity in wound repair can maintain a vicious cycle of chronic inflammation and tissue degradation sustaining a chronic wound. Significantly elevated levels of MMP- 1, − 2 and − 13 in non-healing compared to healing wounds (Fig. 4A) further highlight this point, which is in line with previous reports [14, 32, 39]. For MMP- 2 it was additionally observed that wounds in the inflammatory stage demonstrated significantly elevated levels compared to epithelializing wounds (Fig. 5A), which emphasizes the connection between inflammation and excess proteolysis, reciprocatively maintaining themselves. Later stages of the healing process predominantly demonstrated reduced levels of MMPs, while the regulative counterpart TIMP- 1 displayed increased levels. Also, TIMP- 1 was significantly elevated in AWs as compared to chronic wound entities (except PG, Fig. 3A). Only increased MMP- 7 levels were associated with healing wounds compared to non-healing wounds (Fig. 4A) and continuously increased from an infected/inflammatory stage towards epithelization. While only little is known regarding the role of MMP- 7 in wound healing, it has been reported to be obligatory for the re-epithelization process [16] and was also observed to be elevated in fibrotic processes involving ECM accumulation and activation of TGF beta signaling [45, 46]. This would align with the observed elevated levels in later re-epithelization and remodeling stages indicating healing progression.

Previous work suggested the utilization of the ratio of MMP- 9 and TIMP- 1 as prognostic monitoring parameter for wound healing based on the MMP/TIMP dynamics outlined earlier. Luanraksa et al. (2018) and Li et al. (2013) showed that elevated levels of MMP- 9/TIMP- 1 correlate with a poor healing outcome in DFUs [25, 26]. In our study we also investigated this potential biomarker in terms of discrimination between healing and non-healing and infected vs. non-infected wounds irrespective of entity (Fig. 7). The results align with previous reports, however showing an overall lower AUC than reported in previous studies (0.6124 in our study vs. 0.658 in Li et al., 2013) in terms of healing discrimination. The ratio performed even worse in discriminating infected from inflamed wounds (Figs. 7B and 7 C). Therefore, the ratios value is generally limited, but could potentially aid in differentiating healing from non-healing tendencies in chronic wounds.

A prolonged and dysregulated pro-inflammatory state has been described as a major pathological driver in chronic wounds. The inflammatory response to tissue injury that is generally necessary at the onset can become misdirected, perpetuated, or excessive. This can lead to continuous reactivation and sustained tissue damage, which can in turn trigger a continuous inflammatory response by resident and migrating immune cells [3, 15, 29]. The general human immune response to tissue damage or external pathogens can be categorized in type- 1, type- 2 or type- 3 immune responses, each with specific triggers and response patterns regarding cellular and humoral respondents [47]. In addition to external pathogens such as bacteria or viruses, that can trigger an immune response (pathogen-associated molecular pattern—PAMPs), internal molecules released through (continuous) tissue damage (damage-associated molecular patterns—DAMPs) can trigger an immune response as well [48]. Herein lies an important cornerstone of the vicious cycle that chronic wounds face. Continued tissue damage and local wound infection drive delayed or failed healing. A timely and undisturbed progression through stages of reparative immune response is crucial for tissue repair. The necessary steps, cell types and immune signaling has been extensively described in earlier reviews of experimental data [4, 5, 15, 30]. However, to date, no distinct biomarkers, cellular or humoral diagnostic patterns, made their way into clinical practice. Wound management mainly relies on inaccurate and observer-biased general signs of inflammation (rubor, calor, dolor, tumor, functio laesa), wound bed appearance and wound slough to assess healing status and progress [12, 49]. Analyzing the changing marker patterns in stages of the healing process and understanding the composition of for example wound slough demonstrate potential to objectify assessments [50]. In our study specifically pro-inflammatory interleukins of the IL- 1 family (IL- 1 alpha, IL- 1 beta and IL- 18) demonstrated significantly higher levels in inflammatory stages of the healing process (Fig. 5B), non-healing wounds (Fig. 4B) and infected wounds (Fig. 6B). This underscores the central correlation between excessive inflammation, microbial burden and impaired wound healing. Since IL- 1 family members are a crucial part of the early immune response towards pathogens or damage [47, 51, 52], these cytokines present a significant potential as diagnostic markers to indicate wounds in a highly, potentially excessive, inflammatory state. Generally, IL- 1 family members are released in response to PAMPs and DAMPs by neutrophils, M1-macrophages and resident tissue cells such as fibroblasts or endothelial cells under distress [48, 51–53]. They thereby orchestrate an early and aggressive defensive mechanism of the injured tissue. Further pro-inflammatory cytokines attributed to the initial immune response that are significantly elevated in earlier (infection and inflammation) compared to later wound stages (proliferation and epithelization) in our results were IFN gamma, TNF alpha, G-CSF and GM-CSF (Fig. 5B). All represent relevant signaling agents for inflammation induction, upkeep and regulation, such as M1-macrophage polarization (IFN gamma and TNF alpha [5, 30, 53]), neutrophil differentiation and activation (G-CSF [54] and GM-CSF [55]) or boosting defensive and antimicrobial cellular functions such as phagocytosis, cytokine release and ROS production (GM-CSF [55]). An upregulation during early inflammatory wound stages is to be expected to a certain extend and has been described as part of early type- 1 and type- 3 immune responses [47]. However, in terms of healing progression, IL- 1 alpha, IL- 1 beta, IL- 18 and GM-CSF also demonstrated significantly elevated levels in non-healing wounds in our study (Fig. 4B). This suggests that non-healing wounds fail to progress into a restorative state and maintain a chronic inflammatory state, continuously upholding a harmful microenvironment. Earlier studies observed similar elevated levels of GM-CSF [14] in non-healing wounds and evaluated it to be a potential marker for healing failure in longitudinal analyses. This strengthens GM-CSFs potential as a monitoring parameter for unresolved inflammation jeopardizing healing.

The initial immune response (types 1 and 3) is naturally resolved during the reparative process via suppressive signaling which is generally part of the type 2 immune response. The type 2 response suppresses tissue-damaging effects of sustained or prolonged type 1‑associated inflammation and promotes important tissue repair pathways [47, 56, 57]. This response is majorly driven by the cytokines IL- 4 and IL- 13 released from dendritic cells, innate lymphoid cells (ILC2) and T-helper cells (T_H_2 cells). These induce a phenotypic switch of monocytes and macrophages towards a reparative subset of M2-macrophages crucial for tissue repair (myofibroblast activation) and inflammation resolution by suppressing type 1 immunity [30, 56, 57]. Type 2 immunity in turn can be inhibited by a sustained and excessive pro-inflammatory type 1 response (e.g., excessive IL- 1 beta, IL- 18, IFN gamma) leading to disturbed tissue repair [56, 57].

The natural counterpart of pro-inflammatory IL- 1 family members, IL- 1RA, plays an important regulative role by antagonistically binding to the same receptor as IL- 1 alpha and IL- 1 beta [51, 52, 58, 59]. Preclinical studies in diabetic mouse wound models demonstrated that the signaling of IL- 1R1 and resulting inflammatory effects are tightly regulated by the ratio between IL- 1 beta and IL- 1RA. Impaired wound healing was connected to elevated IL- 1R1 signaling and could be resolved by administering the receptor antagonist IL1-RA in db/db mice [58, 59]. This is reflected in our translational analyses in human wounds by significantly elevated IL- 1RA levels in the epithelization stage of regenerating wounds (Fig. 5B). In earlier inflammatory stages IL- 1RA seems to be upregulated as part of the initial holistic response of the IL- 1 family, however, subsides during the progression from an inflammatory to a proliferative stage. IL- 1RA therefore seems to be a crucial part of the initial inflammatory response as well as the later transition into a reparative state. Based on these results we wondered if a potential ratio of the pro-inflammatory IL- 1 family members to its counterpart IL- 1RA could represent the interconnected dynamics better than its individual markers. Like the previously proposed MMP- 9/TIMP- 1 ratio we investigated the IL- 1 beta/IL- 1RA ratio regarding its ability to indicate a prolonged and excessive inflammatory state as a surrogate for non-healing wounds. As depicted in Fig. 5E, the ratio continuously subsided over the course of healing, illustrating a reduction in IL- 1 beta and/or elevation in IL- 1RA levels during the healing progress. Significantly higher values in non-healing (Fig. 4E), colonized and infected wounds (Fig. 6E) further strengthen its potential as a diagnostic marker for impaired healing. Compared to other ratios, IL- 1 beta/IL- 1RA also demonstrated the best properties to predict a positive healing trajectory with a good AUC of 0.6837 (95%-CI[0.5816; 0.7857; Fig. 7A). Therefore, this newly proposed ratio might be a useful tool to observe excessive unresolved inflammation in chronic wounds as surrogate parameter for failed healing. However, a differentiation between acute infection-triggered or persisting inflammation-triggered elevation of the ratio is not possible, therefore limiting the interpretability to a more general aspect of an elevated immune response. Nonetheless, based on previous pre-clinical studies [58, 59] and our results here, this newly introduced ratio demonstrates potential to be used as a biomarker for persisting chronic inflammation and impaired wound healing. Generally, such markers not only pose diagnostic potential but also options for targeted interventions. In the specific case of IL- 1 family triggered excessive inflammation for example, the use of the recombinant IL- 1 receptor antagonist anakinra could be explored as a potential targeted treatment option for chronic wounds. Prolonged IL- 1R-induced inflammation hindering the wound healing process could be countered. Such approaches are already explored by Tan et al. [58] and Perrault et al. [59] in diabetic mouse models, demonstrating pro-healing results.

To facilitate an adequate immune response, migration of immune cells to the wound site and their activation is vital [5, 27, 47]. Chemotaxis provides the necessary signals to initiate mobilization of precursor and effector cells from the bloodstream and orchestrates the progression of the immune response during regeneration [60]. Basic research into the proceedings of this orderly progression elaborated on the physiological operations and roles of chemokines [27, 60–62]. Classes of chemokines released by resident cells upon tissue damage, such as CXCL8 (IL- 8) [61–63], CCL2 (MCP- 1) [64], CCL3 (MIP- 1 alpha) [65] and CCL4 (MIP- 1 beta) [66] drive inflammatory immune responses. These chemokines recruit and activate effector cells such as neutrophils (CXCL8, CCL3, CCL4), monocytes (CCL2) and macrophages (CCL3, CCL4). Chemokines are further involved in multiple proceedings of the immune response such as macrophage polarization, increased cytokine and chemokines release and leukocyte migration and differentiation [27, 60, 62–64, 67]. For pro-inflammatory chemokines such as CCL2, CCL3, CCL4 and CXCL8, a prolonged activity and repeatedly triggered release in a destruction-activation cycle has been described as part of pathological conditions such as diabetes, atherosclerosis and autoimmune disease [60, 64, 65]. Our results demonstrate similar patterns as pro-inflammatory chemokines CCL2 and CCL3 are significantly elevated in the inflammatory stage compared to the proliferative or epithelization stage (Fig. 5C). CCL3 and CCL4 are significantly increased in non-healing wounds (Fig. 4C), suspected to be caught in an excessive inflammatory state. Also, pro-inflammatory chemokines (CXCL8, CCL2, CCL3, CCL4) were overexpressed in wound entities associated with an increased inflammatory and proteolytic milieu (VLUs, MIXs and DFUs) compared to AWs or WHDs (Fig. 3C). While recruited immune cells are initially necessary for an adequate first-line defense, a more nuanced immune response prone towards regeneration is needed down the line. This is facilitated via phenotype switches in tissue-resident macrophages, circulating monocytes (polarized to M2-macrophages after invasion) and T-lymphocytes of the adaptive immune system (CD4 + and CD8 + lymphocytes) [18, 30, 57, 67]. These cell types employ more targeted defensive and restorative mechanisms compared to the initial response. Therefore, a shift in representative markers can indicate a timely progression from an earlier to a later stage of tissue repair. Apart from cytokines IL- 4 and IL- 13 (which did not demonstrate significant differences in our study), chemokines of the CXCL- 9/− 10/− 11 axis but also CCL3 and CCL4 were shown to induce cellular recruitment of Th1- and Th2-lymphocytes driving the transition towards M2-regenerative macrophage phenotypes [30, 62, 65, 67–69]. Therefore, it needs to be acknowledged that certain chemokines and cytokines attain multiple roles throughout the complex human immune response [4, 54, 55, 60, 64]. However, certain chemokines are more affiliated with either an early pro-inflammatory or later resolving and regenerative response. CXCL8, as a main driver of neutrophil migration and activation in the pro-inflammatory response [63, 67] and CXCL10 (IP- 10), as part of the regulatory response for inflammation resolution and tissue restoration [61, 67, 68, 70] are such counterparts. CXCL10 proved to be one of only two markers to be significantly increased in healing wounds compared to non-healing wounds (Fig. 4C) and showed a continuous median increase with progressing regenerative stages (Fig. 5C). Also, contrary to most other immunomarkers, non-infected wounds demonstrated a significantly higher level of CXCL10 compared to infected wounds (Fig. 6C). Based on these observations we investigated the combination of this pro-healing marker with its appropriate counterpart CXCL8. The CXCL8/CXCL10 ratio proved significantly elevated in non-healing compared to healing wounds (Fig. 4E) and demonstrated a moderate AUC of 0.6530 (95%-CI[0.5515; 0.7545]; Fig. 7A). Its highest potential was observed for infection differentiation with a significantly higher level in infected wounds compared to non-infected and colonized wounds (Fig. 6E) and a very good AUC of 0.7669 (95%-CI[0.6579; 0.8758]; Fig. 7B). As the differentiation between levels of microbial burden and the cut-off to an actual local infection is sometimes difficult and misleading in clinical practice [12, 49], a biomarker that can aid in the distinction would be very valuable. This is especially true if a distinction between infection and inflammation is necessary and if patients present abnormal clinical infection/inflammation signs which is regularly the case in patients with diabetic foot ulcers or immunosuppressive diseases and treatment. A biomarker such as the CXCL8/CXCL10 ratio could support therapeutic decisions regarding the necessity of infection-targeted or inflammation-targeted treatment. Such an informed, targeted approach would greatly help in reducing the unnecessary use of local antiseptic treatments as well as systemic antibiotics in only colonized or inflamed but not actually infected wounds. As the antimicrobial effect of local antiseptics is always accompanied by a certain degree of cytotoxicity, a more nuanced local antiseptic treatment approach could reduce this side-effect and its negative impact on wound healing. We therefore also investigated the ratios’ ability to differentiate between an infection and inflammation stage and found a significantly higher ratio in infected wounds compared to wounds in the inflammation or proliferation stage (Fig. 5E). The CXCL8/CXCL10 ratio was thereby the only tested ratio that demonstrated significant differences between the healing stages and showed a clearly superior AUC of 0.7298 (95%-CI[0.5963; 0.8634], Fig. 7C) for the discrimination between an infected and an inflamed wound. Of all investigated ratios the newly proposed CXCL8/CXCL10 ratio demonstrated significant results for all evaluated clinical outcomes and therefore has a high potential as a diagnostic marker in wound management.

The therapeutic potential of immunomarker monitoring extends beyond the identification of biomarkers; it also encompasses the development of novel treatment modalities. For instance, the use of collagen-based matrices has shown promise in enhancing wound healing through marker analysis, which identified key proteins involved in tissue repair [71]. Especially leveraging the potential of immunomodulatory approaches to inhibit the escalation from an adequately regulated immune response to chronic inflammatory dysregulation could be interesting in the future. Using for example antimicrobial peptides (AMPs) in wound infection treatment strategies or addressing fundamental imbalances by targeting activated damage-associated molecular patterns (DAMPs) or their counterparts suppressing/inhibiting DAMPs (SAMPs) in terms of inflammation resolution, could represent game-changing approaches in the treatment of chronic non-healing wounds [67, 72–74]. Especially the employment of modern materials such as nanoparticles, 3D-printed matrices or bio-scaffolds with or without active agent loading and smart dressings with integrated sensors for diagnostic analyte measurements can open new venues and innovative ways of next-generation wound management [9]. Also, the role of extracellular vesicles (EVs) in wound healing has garnered significant attention in recent years. EVs, which carry a cargo of proteins, lipids, and RNAs, play a crucial role in intercellular communication and tissue repair. Proteomic analyses of EVs derived from mesenchymal stem cells (MSCs) have demonstrated their potential to enhance tissue regeneration by delivering bioactive molecules that promote cell proliferation, migration, and angiogenesis [75, 76]. The therapeutic application of MSC-derived EVs in wound healing is particularly promising, as they can modulate the inflammatory response and facilitate tissue repair in both acute and chronic wound contexts [75, 76]. However, the base of any individualized, targeted, modern therapeutic strategy is the knowledge of underlying dysregulated processes, which represent the targets of correcting interventions. Also, such therapeutic interventions and their success or failure need to be monitored which highlights the importance of exploratory biomarker studies such as the presented work.

To the best of our knowledge, this study is one of the largest and most comprehensive analyses of immunomarker levels and patterns in human chronic wounds so far. Our results expand current knowledge of MMP dynamic in tissue repair, pro- and anti-inflammatory cytokine and chemokine expression and growth factor levels which are thus far predominantly derived from *in-vitro* and animal models. To translate the current knowledge base into clinical usability, such analyses in human cohorts are necessary to isolate potential marker patterns and validate preclinical findings. Our work paints a holistic picture of the physiological and pathophysiological state in various entities, correlates marker levels with relevant clinical outcome parameters and adds new insights regarding less investigated markers in wound healing (MMP- 7). This work also evaluates previously proposed (MMP- 9/TIMP- 1 ratio) and new potential biomarkers (IL- 1 beta/IL- 1RA and CXCL8/CXCL10 ratio).

Naturally, several limitations need to be considered. Data interpretation and extrapolation must be conducted with care. As the current study is a prospective, cross-sectional observation that correlates marker levels to clinical outcomes, predictive relations of investigated markers and results are exploratory at this stage. Prospective, longitudinal case–control studies with larger per group cohorts and ultimately rigorous diagnostic test studies are needed to provide confirmatory results. Thereby, more defined and specified endpoints regarding healing evaluation such as complete wound epithelization over time or area change from baseline need to be employed. Generally, as biomarker studies are difficult to reproduce in diverging scenarios and circumstances, more studies in human cohorts with robust and comparable methodology are needed to validate our primary findings. Establishing multi-center biobanking and registry approaches as in other biomedical research fields, would be valuable. The multicenter umbrella project (Wound-BIOME) of this study emphasizes these necessities. Therefore, joined efforts are currently under way to realize such structures.

## Conclusions

In conclusion, the presented study comprehensively presents immunomarker patterns in chronic wounds and thereby successfully reproduces proposed and observed concepts from *in-vitro* and animal models in a translational approach in the human wound-microenvironment. Chronic non-healing wounds thereby depict an excessive expression of matrix-metalloproteases, pro-inflammatory cytokines and chemokines underscoring the concept of chronic, prolonged inflammation in impaired wound healing. New proposed biomarker ratios for predicting impaired healing based on sustained increased inflammatory activation (IL- 1 beta/IL- 1RA and CXCL8/CXCL10) and distinction between infection-triggered and inflammation-triggered healing impairment (CXCL8/CXCL10) show promising potential to be used in targeted wound care and should be further investigated in future studies.

## Supplementary Information

Supplementary Material 1

Supplementary Material 2

## Data Availability

All data generated or analyzed during this study are included in this published article (and its supplementary information files), raw data are available from the corresponding author on reasonable request.

## Abbreviations

ALU: Arterial leg ulcer
AUC: Area under the curve
AW: Acute wound
CCD: Charge-coupled device
CCL: Chemokine (C–C motif) ligand
CD: Cluster of differentiation
CI: Confidence interval
COL: Colonized
CXCL: Chemokine (C-X-C motif) ligand
DAMP: Damage-associated molecular patterns
DFU: Diabetic foot ulcer
ECM: Extracellular matrix
EGF: Endothelial growth factor
EPITH: Epithelization
FGF: Fibroblast growth factor
G-CSF: Granulocyte colony-stimulating factor
GM-CSF: Granulocyte–macrophage colony-stimulating factor
HEAL: Healing
IFN: Interferon
IL: Interleukin
INF: Infected
INFE: Nfection
INFLA: Inflammation
IQR: Interquartile range
MCP: Monocyte chemoattractant protein
MFI: Mean fluorescence intensity
MIP: Macrophage inflammatory protein
MIX: Arterio-venous/mixed ulcer
MMP: Matrix-metalloprotease
N-HEAL: Non-healing
N-INF: Non-infected
PAMP: Pathogenn-associated molecular pattern
PBS: Phosphate-buffered saline
PDGF: Platelet-derived growth factor
PG: Pyoderma gangrenosum
PROL: Proliferation
QoL: Quality of life
ROC: Receiver-operator curve
rpm: Rounds per minute
RT: Room temperature
SAPE: Streptavidin–phycoerythrin
SD: Standard deviation
TGF: Transforming growth factor
TIMP: Tissue-inhibitor of matrix-metalloprotease
TNF: Tumor necrosis factor
UAB: Universal assay buffer
VEGF: Vascular endothelial growth factor
VLU: Venous leg ulcer
WB: Washing buffer
WHD: Wound healing disorder
