# Supplementary material for: Immunomarker profiling in human chronic wound swabs reveals IL-1 beta/IL-1RA and CXCL8/CXCL10 ratios as potential biomarkers for wound healing, infection status and regenerative stage
Source: J Transl Med. 2025 Apr 8;23:407. doi: 10.1186/s12967-025-06417-2 (PMC11978031; doi:10.1186/s12967-025-06417-2)
Supplement: Supplementary file 1 — Supplementary Material 1 [file 12967_2025_6417_MOESM1_ESM.docx]

**Supplementary Table 1 – Clinical variables assessed by wound care practitioner for each patient in the cohort.**

‘Status’ represents the current healing trajectory (H – healing or NH – non-healing) as assessed by the clinician. ‘Stage’ represents the clinical evaluation of current wound healing stage (INFE – infection, INFL – inflammation, PROL – proliferation or EPITH – epithelization). ‘Infection’ represents the degree of microbial burden (INF – infected, COL – colonized, N-INF – non-infected).

| ID | Center | Entity | Status | Stage | Infection |
| --- | --- | --- | --- | --- | --- |
| P003 | UWH | DFU | NH | INFE | INF |
| P004 | UWH | ALU | NH | INFL | COL |
| P008 | UWH | ALU | NH | INFE | INF |
| P009 | UWH | ALU | NH | PROL | COL |
| P010 | UWH | ALU | H | PROL | N-INF |
| P011 | UWH | ALU | NH | INFE | INF |
| P012 | UWH | ALU | NH | INFE | INF |
| P013 | UWH | VLU | NH | INFE | INF |
| P014 | UWH | VLU | NH | INFL | COL |
| P015 | UWH | ALU | NH | INFL | COL |
| P018 | UWH | ALU | NH | PROL | COL |
| P019 | UWH | VLU | NH | INFL | COL |
| P020 | UWH | VLU | NH | INFL | N-INF |
| P021 | UWH | WHD | H | PROL | N-INF |
| P022 | UWH | VLU | NH | PROL | COL |
| P023 | UWH | MIX | NH | INFL | COL |
| P024 | UWH | ALU | NH | INFL | N-INF |
| P027 | UKE | WHD | NH | EPITH | N-INF |
| P028 | UKE | MIX | NH | INFL | COL |
| P029 | UKE | ALU | H | EPITH | N-INF |
| P030 | UKE | VLU | H | INFL | COL |
| P031 | UKE | VLU | NH | INFL | COL |
| P032 | UKE | VLU | NH | INFE | INF |
| P033 | UKE | ALU | NH | INFL | COL |
| P034 | UKE | MIX | NH | INFL | COL |
| P035 | UKE | PG | H | EPITH | N-INF |
| P036 | UKE | PG | NH | INFE | INF |
| P037 | UKE | PG | NH | INFL | COL |
| P038 | UKE | ALU | NH | INFL | COL |
| P039 | UKES | ALU | NH | INFL | COL |
| P040 | UKES | PG | NH | INFL | COL |
| P041 | UKES | WHD | H | EPITH | N-INF |
| P042 | UKES | VLU | NH | INFL | COL |
| P043 | UKES | MIX | NH | INFL | COL |
| P044 | UKES | VLU | NH | INFL | COL |
| P045 | UKES | WHD | H | EPITH | COL |
| P046 | UKES | PG | H | PROL | N-INF |
| P047 | UKES | PG | H | PROL | COL |
| P048 | UKES | VLU | NH | INFL | COL |
| P049 | UKES | VLU | NH | PROL | COL |
| P050 | UKES | MIX | NH | PROL | COL |
| P051 | UKES | VLU | NH | INFL | COL |
| P052 | UKES | VLU | H | EPITH | COL |
| P053 | UKES | WHD | H | EPITH | COL |
| P055 | UKES | WHD | H | PROL | COL |
| P057 | UKES | PG | H | PROL | COL |
| P058 | UKES | VLU | NH | INFE | INF |
| P060 | UKES | WHD | H | PROL | COL |
| P061 | UKES | WHD | NH | INFL | N-INF |
| P062 | UKES | WHD | H | EPITH | N-INF |
| P063 | UKES | VLU | H | PROL | COL |
| P064 | UKES | WHD | H | PROL | COL |
| P065 | UKES | WHD | H | PROL | N-INF |
| P066 | UKES | WHD | NH | PROL | COL |
| P067 | UKES | VLU | H | EPITH | N-INF |
| P068 | UKES | VLU | NH | PROL | N-INF |
| P069 | UKES | WHD | NH | EPITH | N-INF |
| P071 | UKES | WHD | NH | PROL | COL |
| P072 | UKES | PG | NH | INFL | COL |
| P073 | UKES | VLU | H | PROL | N-INF |
| P074 | UKES | VLU | NH | PROL | COL |
| P075 | UKES | VLU | H | PROL | N-INF |
| P076 | UKES | WHD | H | EPITH | N-INF |
| P077 | UKES | VLU | NH | INFL | COL |
| P078 | UKES | DFU | H | PROL | N-INF |
| P079 | UKES | VLU | NH | INFL | COL |
| P081 | UKES | VLU | NH | PROL | N-INF |
| P082 | UKES | WHD | H | EPITH | N-INF |
| P083 | UKES | VLU | NH | INFL | COL |
| P084 | UKES | WHD | H | PROL | COL |
| P086 | UKES | WHD | NH | INFL | COL |
| P087 | UKES | MIX | NH | INFL | COL |
| P088 | UKES | WHD | NH | INFL | COL |
| P089 | UKE | PG | NH | INFL | COL |
| P090 | UKE | VLU | H | EPITH | N-INF |
| P092 | UKE | WHD | NH | EPITH | N-INF |
| P095 | UKE | PG | NH | INFL | COL |
| P096 | UKE | VLU | NH | INFE | INF |
| P097 | UWH | AW | H | PROL | N-INF |
| P098 | UWH | AW | H | PROL | N-INF |
| P099 | UWH | AW | H | PROL | N-INF |
| P100 | UWH | AW | H | PROL | N-INF |
| P101 | UWH | AW | H | PROL | N-INF |
| P102 | UWH | AW | H | PROL | N-INF |
| P103 | UWH | AW | H | PROL | N-INF |
| P104 | UKD | ALU | NH | INFE | INF |
| P105 | UKD | VLU | H | PROL | COL |
| P106 | UKD | ALU | H | PROL | N-INF |
| P107 | UKD | DFU | NH | INFL | COL |
| P108 | UKD | DFU | H | PROL | N-INF |
| P110 | UKD | DFU | H | EPITH | N-INF |
| P111 | UKD | DFU | NH | INFL | COL |
| P112 | UKD | DFU | H | PROL | N-INF |
| P113 | UKE | PG | NH | INFE | INF |
| P115 | UKE | VLU | H | INFL | N-INF |
| P124 | UKE | MIX | NH | INFL | COL |
| P125 | UKE | MIX | NH | INFL | COL |
| P129 | UKE | VLU | H | INFL | COL |
| P130 | UKE | PG | NH | PROL | COL |
| P141 | UKD | DFU | NH | INFL | N-INF |
| P142 | UKD | DFU | H | PROL | COL |
| P143 | UKD | DFU | H | INFL | N-INF |
| P144 | UKD | DFU | H | EPITH | COL |
| P145 | UKD | DFU | NH | INFE | INF |
| P146 | UKD | DFU | NH | INFL | COL |
| P147 | UKD | DFU | NH | INFE | INF |
| P148 | UKD | DFU | NH | INFL | COL |
| P149 | UKD | AW | H | PROL | N-INF |
| P150 | UKD | AW | H | PROL | N-INF |
| P151 | UKD | AW | H | PROL | N-INF |

*(ALU – arterial leg ulcer, AW – acute wound, DFU – diabetic foot ulcer, VLU – venous leg ulcer, PG – pyoderma gangrenosum, WHD – wound healing disorder, MIX – mixed leg ulcer;* *UWH - Witten/Herdecke University, UKE - University Medical Center Hamburg-Eppendorf, UKES - University Medical Center Essen, UKD - University Medical Center Duesseldorf)*
