# Supplementary material for: Immunomarker profiling in human chronic wound swabs reveals IL-1 beta/IL-1RA and CXCL8/CXCL10 ratios as potential biomarkers for wound healing, infection status and regenerative stage
Source: J Transl Med. 2025 Apr 8;23:407. doi: 10.1186/s12967-025-06417-2 (PMC11978031; doi:10.1186/s12967-025-06417-2)
Supplement: Supplementary file 2 — Supplementary Material 2 [file 12967_2025_6417_MOESM2_ESM.docx]

**Supplementary Table 2 – Summary measurement results for analytes allocated to different clinical outcome measurements.**

The table summarizes the measurement results of the quantification of all 35 investigated analytes for the different explored outcome measurements: healing status (A), entity differentiation (B), stage differentiation (C) and clinical microbial burden (D). Mean (M), median (MD), standard deviation (SD), standard error of the mean (SEM) as well as range (MIN/MAX) and number of cases per outcome (n) are reported. Results are depicted in pg/ml and p-values for all comparisons are stated with and α-level of 5% (*p* < 0.05). Where appropriate, a multiple comparisons adjustment was performed.

1. Healing status (H – healing, NH – non-healing)

|  | Non-Healing | | | | | | Healing | | | | | | p-value* |
| --- | --- | --- | --- | --- | --- | --- | --- | --- | --- | --- | --- | --- | --- |
|  | **M** | **MD** | **SD** | **MIN** | **MAX** | **n** | **M** | **MD** | **SD** | **MIN** | **MAX** | **n** |  |
| MMP-1 | 9005.00 | 3819.00 | 12884.00 | 66.19 | 40400.00 | 46 | 13829.00 | 6692.00 | 15806.00 | 247.20 | 64850.00 | 64 | **0.0344 (*)** |
| MMP-2 | 55.68 | 31.85 | 91.44 | 4.52 | 598.20 | 46 | 115.70 | 60.20 | 153.40 | 3.27 | 880.40 | 64 | **0.0195 (*)** |
| MMP-3 | 209.00 | 134.80 | 213.90 | 9.96 | 1071.00 | 46 | 298.80 | 152.10 | 517.60 | 15.20 | 3664.00 | 64 | 0.5270 |
| MMP-7 | 440.80 | 288.20 | 508.80 | 11.87 | 2493.00 | 46 | 685.80 | 195.40 | 2338.00 | 21.61 | 17555.00 | 64 | **0.0354 (*)** |
| MMP-8 | 27017.00 | 20290.00 | 24142.00 | 700.20 | 126200.00 | 46 | 35382.00 | 26700.00 | 30118.00 | 2567.00 | 126200.00 | 64 | 0.0730 |
| MMP-9 | 1553.00 | 884.40 | 2625.00 | 18.35 | 13375.00 | 46 | 4574.00 | 1029.00 | 16733.00 | 15.44 | 130095.00 | 64 | 0.2254 |
| MMP-12 | 581.20 | 188.90 | 1012.00 | 4.20 | 5606.00 | 46 | 747.10 | 217.20 | 1405.00 | 0.47 | 6158.00 | 64 | 0.2932 |
| MMP-13 | 136.60 | 17.09 | 413.30 | 3.39 | 2730.00 | 46 | 220.50 | 46.99 | 483.10 | 0.85 | 3141.00 | 64 | **0.0127 (*)** |
| TIMP-1 | 5159.00 | 2135.00 | 6920.00 | 62.40 | 26450.00 | 46 | 2863.00 | 1918.00 | 3095.00 | 102.10 | 15495.00 | 64 | 0.5880 |
| IL-1 alpha | 268.60 | 184.70 | 361.00 | 4.13 | 2119.00 | 46 | 480.80 | 252.90 | 781.40 | 9.29 | 4574.00 | 64 | **0.0376 (*)** |
| IL-1 beta | 4457.00 | 1586.00 | 7744.00 | 4.90 | 36995.00 | 46 | 10207.00 | 3637.00 | 18511.00 | 42.77 | 110350.00 | 64 | **0.0022 (*)** |
| IL-1RA | 361741.00 | 73060.00 | 1211391.00 | 358.90 | 8002000.00 | 46 | 293695.00 | 105645.00 | 915066.00 | 1070.00 | 7209000.00 | 64 | 0.3494 |
| IL-4 | 56.83 | 15.74 | 82.27 | 1.48 | 405.50 | 46 | 75.12 | 21.12 | 109.40 | 7.07 | 486.40 | 64 | 0.3250 |
| IL-6 | 11982.00 | 3702.00 | 20521.00 | 4.73 | 104270.00 | 46 | 14708.00 | 4737.00 | 23091.00 | 66.20 | 135500.00 | 64 | 0.2936 |
| IL-9 | 54.97 | 52.27 | 27.31 | 5.66 | 108.60 | 46 | 64.75 | 67.05 | 33.12 | 1.00 | 131.50 | 64 | 0.1065 |
| IL-10 | 13.06 | 3.83 | 19.27 | 0.31 | 93.84 | 46 | 18.82 | 5.89 | 40.54 | 0.21 | 303.70 | 64 | 0.4003 |
| IL-13 | 5.38 | 4.40 | 4.43 | 0.33 | 19.73 | 46 | 7.19 | 4.40 | 7.81 | 0.60 | 38.17 | 64 | 0.6026 |
| IL-17A | 91.23 | 26.52 | 221.40 | 1.53 | 1476.00 | 46 | 82.10 | 33.53 | 120.90 | 2.26 | 571.10 | 64 | 0.4209 |
| IL-18 | 291.40 | 190.20 | 361.20 | 13.03 | 1959.00 | 46 | 878.30 | 308.00 | 1844.00 | 25.22 | 1959.00 | 64 | **0.0056 (*)** |
| IL-21 | 170.60 | 93.96 | 174.80 | 16.89 | 747.10 | 46 | 232.70 | 104.90 | 303.60 | 15.23 | 1426.00 | 64 | 0.5251 |
| IL-8 (CXCL8) | 8455.00 | 3253.00 | 14312.00 | 407.60 | 77120.00 | 46 | 204787.00 | 3722.00 | 1579111.00 | 339.80 | 12640000.00 | 64 | 0.5797 |
| IP-10 (CXCL10) | 227.20 | 98.41 | 316.70 | 1.67 | 1563.00 | 46 | 204.30 | 33.08 | 546.10 | 2.33 | 3805.00 | 64 | **0.0070 (*)** |
| MCP-1 (CCL2) | 1753.00 | 1348.00 | 1590.00 | 26.97 | 6858.00 | 46 | 1946.00 | 1404.00 | 1875.00 | 22.34 | 7256.00 | 64 | 0.9399 |
| MIP-1 alpha (CCL3) | 421.90 | 290.80 | 473.30 | 11.59 | 1869.00 | 46 | 837.10 | 493.30 | 1023.00 | 22.35 | 5288.00 | 64 | **0.0073 (*)** |
| MIP-1 beta (CCL4) | 873.10 | 455.40 | 1028.00 | 43.12 | 4791.00 | 46 | 2026.00 | 844.70 | 3588.00 | 8.06 | 19735.00 | 64 | **0.0328 (*)** |
| IFN gamma | 84.10 | 51.12 | 83.22 | 7.18 | 422.50 | 46 | 156.70 | 96.04 | 181.40 | 6.76 | 891.30 | 64 | 0.0591 |
| TGF alpha | 33.53 | 18.63 | 42.86 | 0.69 | 202.40 | 46 | 42.28 | 19.17 | 58.67 | 1.38 | 343.20 | 64 | 0.2162 |
| TNF alpha | 169.70 | 170.60 | 125.30 | 2.58 | 539.70 | 46 | 278.90 | 215.60 | 289.00 | 8.81 | 1443.00 | 64 | 0.0683 |
| TNF beta | 29.82 | 6.86 | 99.00 | 2.58 | 570.40 | 46 | 75.35 | 6.86 | 352.60 | 4.46 | 2734.00 | 64 | 0.3964 |
| G-CSF | 648.80 | 75.37 | 1090.00 | 7.43 | 4414.00 | 46 | 890.20 | 150.10 | 1402.00 | 10.00 | 6548.00 | 64 | 0.0566 |
| GM-CSF | 59.35 | 44.19 | 61.12 | 5.80 | 313.50 | 46 | 105.70 | 67.61 | 145.90 | 8.07 | 876.00 | 64 | **0.0137 (*)** |
| FGF-2 | 434.50 | 88.96 | 1216.00 | 1.82 | 8121.00 | 46 | 467.60 | 156.30 | 989.50 | 2.73 | 7026.00 | 64 | 0.1165 |
| EGF | 7.53 | 5.28 | 7.21 | 0.77 | 29.82 | 46 | 9.89 | 6.90 | 9.93 | 1.17 | 66.12 | 64 | 0.0714 |
| PDGF-BB | 78.56 | 32.40 | 113.10 | 2.45 | 503.20 | 46 | 72.74 | 48.35 | 104.50 | 7.31 | 759.30 | 64 | 0.4752 |
| VEGF-A | 4846.00 | 3429.00 | 6131.00 | 225.40 | 36575.00 | 46 | 6134.00 | 5084.00 | 4503.00 | 228.20 | 17805.00 | 64 | **0.0232 (*)** |

*(MMP – matrix-metalloprotease, TIMP – tissue-inhibitor-of-matrix-metalloprotease, IL – interleukin, IP – interferon gamma-induced protein, MCP – monocyte chemoattractant protein, MIP – macrophage inflammatory protein, IFN – interferon,* *TGF – transforming growth factor, TNF – tumor necrosis factor, G-CSF – granulocyte colony stimulating factor, GM-CSF – granulocyte macrophage-colony stimulating factor, FGF – fibroblast growth factor, EGF – epidermal growth factor, PDGF – platelet-derived growth factor, VEGF – vascular endothelial growth factor)*

1. Entities (AW – acute wounds; WHD – wound healing disorder; ALU – arterial leg ulcer; VLU – venous leg ulcer; DFU – diabetic foot ulcer; MIX – mixed arterio-venous ulcer; PG – pyoderma gangrenosum)

|  |  | MMP-1 | MMP-2 | MMP-3 | MMP-7 | | MMP-8 | | MMP-9 | | MMP-12 | MMP-13 | TIMP-1 | IL-1 alpha | IL-1  beta | IL-1RA | IL-4 | IL-6 |
| --- | --- | --- | --- | --- | --- | --- | --- | --- | --- | --- | --- | --- | --- | --- | --- | --- | --- | --- |
| AW | **n** | 10 | 10 | 10 | 10 | | 10 | | 10 | | 10 | 10 | 10 | 10 | 10 | 10 | 10 | 10 |
|  | **MD** | 1501.00 | 40.92 | 453.30 | 336.20 | | 7486.00 | | 926.20 | | 4.20 | 7.39 | 16318.00 | 17.43 | 14.40 | 15771.00 | 12.65 | 9775.00 |
|  | **MIN** | 880.70 | 17.71 | 69.77 | 11.87 | | 700.20 | | 37.77 | | 4.20 | 4.58 | 3539.00 | 14.01 | 4.90 | 1280.00 | 7.46 | 1043.00 |
|  | **MAX** | 2973.00 | 141.40 | 1071.00 | 2397.00 | | 15330.00 | | 3462.00 | | 32.97 | 242.00 | 26450.00 | 55.37 | 286.80 | 63045.00 | 120.90 | 104270.00 |
|  | **M** | 1653.00 | 48.46 | 430.70 | 556.30 | | 7789.00 | | 1165.00 | | 10.49 | 37.22 | 15645.00 | 22.23 | 49.27 | 22539.00 | 36.74 | 25885.00 |
|  | **SD** | 648.30 | 36.66 | 286.50 | 683.50 | | 4652.00 | | 1010.00 | | 11.37 | 73.53 | 7849.00 | 12.52 | 87.56 | 22632.00 | 43.20 | 33179.00 |
| WHD | **n** | 20 | 20 | 20 | 20 | | 20 | | 20 | | 20 | 20 | 20 | 20 | 20 | 20 | 20 | 20 |
|  | **MD** | 2440.00 | 7.94 | 27.35 | 180.10 | | 20078.00 | | 322.20 | | 120.00 | 12.69 | 1266.00 | 137.30 | 2078.00 | 92448.00 | 12.24 | 986.60 |
|  | **MIN** | 66.19 | 4.64 | 9.96 | 70.52 | | 6768.00 | | 37.35 | | 4.20 | 3.39 | 87.10 | 4.13 | 38.27 | 642.30 | 7.07 | 4.73 |
|  | **MAX** | 40400.00 | 219.00 | 504.80 | 17555.00 | | 89990.00 | | 13375.00 | | 2120.00 | 1152.00 | 10701.00 | 515.00 | 28595.00 | 7209000.00 | 56.60 | 51100.00 |
|  | **M** | 8490.00 | 37.10 | 114.60 | 1126.00 | | 26288.00 | | 1961.00 | | 284.50 | 77.44 | 2122.00 | 162.60 | 4305.00 | 473860.00 | 13.88 | 5756.00 |
|  | **SD** | 12536.00 | 63.14 | 157.10 | 3873.00 | | 19874.00 | | 3863.00 | | 503.00 | 254.00 | 2545.00 | 152.40 | 7403.00 | 1590606.00 | 10.93 | 13880.00 |
| ALU | **n** | 15 | 15 | 15 | 15 | | 15 | | 15 | | 15 | 15 | 15 | 15 | 15 | 15 | 15 | 15 |
|  | **MD** | 7046.00 | 49.26 | 135.70 | 192.70 | | 22050.00 | | 518.50 | | 117.40 | 21.18 | 1220.00 | 113.00 | 1977.00 | 57995.00 | 18.09 | 3942.00 |
|  | **MIN** | 602.30 | 3.27 | 16.70 | 47.41 | | 10953.00 | | 191.60 | | 0.47 | 5.93 | 596.40 | 43.46 | 113.30 | 12040.00 | 7.46 | 156.00 |
|  | **MAX** | 40400.00 | 165.20 | 551.60 | 2493.00 | | 126200.00 | | 8595.00 | | 1728.00 | 709.50 | 7996.00 | 962.70 | 72075.00 | 499950.00 | 236.70 | 51100.00 |
|  | **M** | 13661.00 | 65.77 | 182.10 | 403.10 | | 36654.00 | | 1393.00 | | 295.80 | 121.90 | 2424.00 | 212.10 | 7201.00 | 97139.00 | 42.66 | 9713.00 |
|  | **SD** | 15008.00 | 55.12 | 152.60 | 637.20 | | 35282.00 | | 2191.00 | | 450.00 | 209.50 | 2365.00 | 238.10 | 18082.00 | 135499.00 | 68.51 | 13784.00 |
| VLU | **n** | 30 | 30 | 30 | 30 | | 30 | | 30 | | 30 | 30 | 30 | 30 | 30 | 30 | 30 | 30 |
|  | **MD** | 8487.00 | 69.47 | 167.90 | 254.90 | | 32593.00 | | 1473.00 | | 376.30 | 50.00 | 2151.00 | 302.70 | 4316.00 | 157350.00 | 15.22 | 5913.00 |
|  | **MIN** | 191.20 | 4.52 | 15.20 | 56.27 | | 6472.00 | | 18.35 | | 3.42 | 2.64 | 62.40 | 10.71 | 53.25 | 358.90 | 2.64 | 43.25 |
|  | **MAX** | 50460.00 | 880.40 | 3664.00 | 6307.00 | | 126200.00 | | 130095.00 | | 6158.00 | 2730.00 | 15495.00 | 2119.00 | 110350.00 | 1984500.00 | 405.50 | 51100.00 |
|  | **M** | 18473.00 | 145.40 | 407.00 | 639.30 | | 43367.00 | | 8161.00 | | 1186.00 | 212.80 | 3382.00 | 416.50 | 12990.00 | 373955.00 | 38.97 | 14163.00 |
|  | **SD** | 17671.00 | 203.40 | 698.60 | 1320.00 | | 32793.00 | | 24055.00 | | 1868.00 | 523.60 | 3820.00 | 406.30 | 22088.00 | 482466.00 | 78.27 | 18781.00 |
| DFU | **n** | 15 | 15 | 15 | 15 | | 15 | | 15 | | 15 | 15 | 15 | 15 | 15 | 15 | 15 | 15 |
|  | **MD** | 6017.00 | 30.30 | 166.60 | 182.40 | | 21155.00 | | 675.70 | | 241.10 | 340.40 | 1564.00 | 205.00 | 3450.00 | 108390.00 | 120.90 | 3768.00 |
|  | **MIN** | 1243.00 | 5.95 | 20.55 | 21.61 | | 2567.00 | | 15.44 | | 8.82 | 21.56 | 364.00 | 54.27 | 42.77 | 9237.00 | 12.65 | 408.90 |
|  | **MAX** | 40400.00 | 137.10 | 507.60 | 800.70 | | 71220.00 | | 1832.00 | | 2518.00 | 3141.00 | 11894.00 | 3204.00 | 17735.00 | 353650.00 | 486.40 | 135500.00 |
|  | **M** | 8235.00 | 44.78 | 176.80 | 253.00 | | 25714.00 | | 665.00 | | 640.50 | 571.60 | 2383.00 | 426.40 | 4436.00 | 112586.00 | 145.10 | 16260.00 |
|  | **SD** | 9541.00 | 40.66 | 148.30 | 217.00 | | 19410.00 | | 631.50 | | 803.20 | 818.30 | 2818.00 | 779.00 | 5562.00 | 87971.00 | 117.70 | 34366.00 |
| MIX | **n** | 8 | 8 | 8 | 8 | | 8 | | 8 | | 8 | 8 | 8 | 8 | 8 | 8 | 8 | 8 |
|  | **MD** | 8877.00 | 214.40 | 159.60 | 142.80 | | 28918.00 | | 1013.00 | | 981.20 | 66.88 | 1668.00 | 1033.00 | 7852.00 | 180093.00 | 286.60 | 17936.00 |
|  | **MIN** | 1798.00 | 20.66 | 32.70 | 49.24 | | 16310.00 | | 117.00 | | 212.60 | 16.23 | 591.80 | 643.00 | 2167.00 | 98015.00 | 169.30 | 756.00 |
|  | **MAX** | 18130.00 | 511.40 | 1356.00 | 538.50 | | 44925.00 | | 2999.00 | | 5551.00 | 603.30 | 6200.00 | 4574.00 | 13739.00 | 346550.00 | 369.00 | 59435.00 |
|  | **M** | 8957.00 | 219.20 | 300.80 | 219.60 | | 28854.00 | | 1332.00 | | 1620.00 | 158.70 | 2098.00 | 1697.00 | 8306.00 | 188309.00 | 275.30 | 23402.00 |
|  | **SD** | 5305.00 | 158.30 | 434.10 | 171.20 | | 9953.00 | | 914.90 | | 1873.00 | 201.90 | 1782.00 | 1405.00 | 4052.00 | 85595.00 | 60.57 | 22479.00 |
| PG | **n** | 12 | 12 | 12 | 12 | | 12 | | 12 | | 12 | 12 | 12 | 12 | 12 | 12 | 12 | 12 |
|  | **MD** | 4866.00 | 46.87 | 162.30 | 333.50 | | 36368.00 | | 1852.00 | | 257.00 | 16.62 | 2459.00 | 327.90 | 6056.00 | 162575.00 | 22.51 | 5268.00 |
|  | **MIN** | 492.60 | 6.25 | 22.90 | 48.13 | | 5806.00 | | 103.30 | | 4.20 | 0.85 | 354.10 | 5.70 | 130.90 | 496.40 | 1.48 | 77.09 |
|  | **MAX** | 43805.00 | 309.00 | 444.00 | 1448.00 | | 126200.00 | | 8204.00 | | 1698.00 | 171.50 | 6053.00 | 683.60 | 55775.00 | 8002000.00 | 181.10 | 51100.00 |
|  | **M** | 11469.00 | 80.43 | 178.30 | 442.00 | | 36352.00 | | 2244.00 | | 512.40 | 34.44 | 2612.00 | 333.70 | 11749.00 | 822934.00 | 49.15 | 9735.00 |
|  | **SD** | 15009.00 | 89.52 | 131.50 | 422.40 | | 32127.00 | | 2112.00 | | 586.80 | 47.69 | 1467.00 | 233.40 | 16062.00 | 2264900.00 | 63.83 | 14085.00 |
| p-value | | **0.0101**  **(*)** | **0.0011**  **(*)** | **0.0070**  **(*)** | 0.5017 | **0.0004**  **(*)** | | **0.0051**  **(*)** | | **0.0001**  **(*)** | | **<0.0001**  **(*)** | **0.0002**  **(*)** | **<0.0001**  **(*)** | **<0.0001**  **(*)** | **0.0011**  **(*)** | **<0.0001**  **(*)** | **0.0057**  **(*)** |

|  |  | IL-9 | IL-10 | IL-13 | IL-17A | IL-18 | IL-21 | IFN gamma | G-CSF | GM-CSF | TNF alpha | TNF  beta | TGF alpha | IL-8 (CXCL8) | IP-10 (CXCL10) |
| --- | --- | --- | --- | --- | --- | --- | --- | --- | --- | --- | --- | --- | --- | --- | --- |
| AW | **n** | 10 | 10 | 10 | 10 | 10 | 10 | 10 | 10 | 10 | 10 | 10 | 10 | 10 | 10 |
|  | **MD** | 28.45 | 5.21 | 4.40 | 6.40 | 120.70 | 72.87 | 37.04 | 46.41 | 18.43 | 208.70 | 6.86 | 4.40 | 1398.00 | 72.94 |
|  | **MIN** | 17.86 | 0.49 | 4.40 | 2.81 | 26.60 | 45.18 | 7.18 | 20.17 | 8.07 | 54.45 | 6.13 | 2.72 | 600.60 | 7.21 |
|  | **MAX** | 66.66 | 93.84 | 7.49 | 72.53 | 213.60 | 431.90 | 96.38 | 1593.00 | 58.16 | 284.50 | 6.86 | 7.47 | 4710.00 | 617.20 |
|  | **M** | 35.56 | 25.73 | 4.96 | 17.73 | 124.80 | 145.70 | 45.61 | 211.60 | 27.44 | 197.50 | 6.64 | 4.61 | 1967.00 | 148.30 |
|  | **SD** | 16.43 | 33.08 | 1.05 | 22.23 | 62.21 | 130.50 | 28.62 | 486.40 | 19.83 | 66.71 | 0.35 | 1.17 | 1411.00 | 188.50 |
| WHD | **n** | 20 | 20 | 20 | 20 | 20 | 20 | 20 | 20 | 20 | 20 | 20 | 20 | 20 | 20 |
|  | **MD** | 37.63 | 1.78 | 2.65 | 18.39 | 118.80 | 56.78 | 24.60 | 54.52 | 18.43 | 48.78 | 6.86 | 11.27 | 2717.00 | 41.02 |
|  | **MIN** | 5.66 | 0.31 | 0.63 | 2.26 | 24.25 | 16.89 | 6.76 | 7.43 | 11.22 | 2.58 | 6.86 | 0.69 | 584.40 | 1.67 |
|  | **MAX** | 74.88 | 42.04 | 4.40 | 74.86 | 1570.00 | 116.90 | 391.00 | 1994.00 | 191.80 | 247.50 | 6.86 | 261.50 | 53030.00 | 1550.00 |
|  | **M** | 39.80 | 4.18 | 2.92 | 27.95 | 258.20 | 63.52 | 55.92 | 214.00 | 42.89 | 70.67 | 6.86 | 29.21 | 6055.00 | 158.40 |
|  | **SD** | 19.94 | 9.12 | 1.37 | 25.36 | 388.10 | 30.95 | 84.57 | 468.70 | 52.81 | 66.77 | 0.00 | 57.72 | 11384.00 | 346.50 |
| ALU | **n** | 15 | 15 | 15 | 15 | 15 | 15 | 15 | 15 | 15 | 15 | 15 | 15 | 15 | 15 |
|  | **MD** | 78.12 | 3.09 | 4.40 | 19.61 | 192.10 | 89.44 | 51.12 | 111.20 | 46.33 | 199.30 | 6.86 | 14.81 | 3570.00 | 18.81 |
|  | **MIN** | 40.16 | 1.04 | 2.76 | 6.82 | 73.42 | 50.56 | 18.76 | 41.02 | 8.07 | 22.09 | 2.58 | 1.38 | 1682.00 | 3.11 |
|  | **MAX** | 121.50 | 41.62 | 13.48 | 193.00 | 2278.00 | 515.70 | 225.50 | 3633.00 | 876.00 | 724.70 | 371.90 | 84.37 | 31815.00 | 1287.00 |
|  | **M** | 80.27 | 7.21 | 5.36 | 45.95 | 377.50 | 140.00 | 81.78 | 651.60 | 103.90 | 235.10 | 31.09 | 26.11 | 8175.00 | 217.00 |
|  | **SD** | 20.82 | 11.07 | 3.33 | 58.34 | 558.50 | 144.40 | 68.63 | 1186.00 | 215.80 | 172.50 | 94.29 | 26.94 | 10444.00 | 404.10 |
| VLU | **n** | 30 | 30 | 30 | 30 | 30 | 30 | 30 | 30 | 30 | 30 | 30 | 30 | 30 | 30 |
|  | **MD** | 63.90 | 5.89 | 3.36 | 33.53 | 275.10 | 102.80 | 92.44 | 107.80 | 70.43 | 153.00 | 6.86 | 30.16 | 4876.00 | 53.84 |
|  | **MIN** | 15.05 | 0.21 | 0.60 | 1.53 | 13.03 | 26.30 | 8.42 | 10.00 | 5.80 | 5.10 | 6.86 | 1.01 | 1324.00 | 2.37 |
|  | **MAX** | 120.40 | 82.20 | 19.73 | 1476.00 | 12415.00 | 747.10 | 891.30 | 4414.00 | 334.20 | 896.00 | 570.40 | 202.40 | 12640000.00 | 768.50 |
|  | **M** | 64.19 | 12.29 | 3.98 | 113.10 | 933.20 | 137.90 | 145.40 | 484.60 | 89.63 | 222.10 | 39.72 | 46.05 | 432147.00 | 133.90 |
|  | **SD** | 27.81 | 17.66 | 3.87 | 272.40 | 2273.00 | 146.50 | 185.60 | 907.60 | 74.40 | 202.80 | 121.70 | 48.14 | 2305765.00 | 187.70 |
| DFU | **n** | 15 | 15 | 15 | 15 | 15 | 15 | 15 | 15 | 15 | 15 | 15 | 15 | 15 | 15 |
|  | **MD** | 69.45 | 19.13 | 9.97 | 74.21 | 289.10 | 347.20 | 151.40 | 1541.00 | 63.54 | 255.10 | 6.13 | 24.02 | 6375.00 | 57.47 |
|  | **MIN** | 14.44 | 3.25 | 0.33 | 7.19 | 74.78 | 15.23 | 17.14 | 63.65 | 16.87 | 27.14 | 6.13 | 3.10 | 1620.00 | 3.96 |
|  | **MAX** | 131.50 | 68.32 | 38.17 | 484.60 | 1202.00 | 1092.00 | 705.70 | 6548.00 | 313.50 | 1365.00 | 219.20 | 133.40 | 37995.00 | 944.60 |
|  | **M** | 68.08 | 22.76 | 11.01 | 99.46 | 381.20 | 335.70 | 156.30 | 1648.00 | 87.82 | 295.20 | 25.83 | 31.04 | 8543.00 | 208.70 |
|  | **SD** | 35.56 | 17.74 | 8.87 | 117.80 | 309.40 | 281.50 | 164.40 | 1746.00 | 87.28 | 314.10 | 55.38 | 32.91 | 9249.00 | 333.80 |
| MIX | **n** | 8 | 8 | 8 | 8 | 8 | 8 | 8 | 8 | 8 | 8 | 8 | 8 | 8 | 8 |
|  | **MD** | 77.81 | 36.99 | 21.76 | 254.90 | 928.80 | 774.60 | 351.90 | 2831.00 | 157.70 | 587.80 | 19.70 | 82.00 | 4896.00 | 187.30 |
|  | **MIN** | 23.86 | 13.32 | 13.48 | 138.60 | 436.80 | 449.20 | 168.40 | 1457.00 | 65.96 | 279.50 | 6.13 | 20.28 | 1236.00 | 13.29 |
|  | **MAX** | 120.30 | 303.70 | 33.49 | 571.10 | 3124.00 | 1426.00 | 630.20 | 6034.00 | 752.80 | 1443.00 | 2734.00 | 343.20 | 8197.00 | 3805.00 |
|  | **M** | 76.12 | 69.05 | 21.70 | 291.20 | 1434.00 | 843.90 | 354.50 | 2974.00 | 221.60 | 668.80 | 448.30 | 112.20 | 4908.00 | 701.60 |
|  | **SD** | 29.56 | 95.99 | 5.99 | 145.50 | 1101.00 | 299.50 | 144.30 | 1428.00 | 220.60 | 360.10 | 950.50 | 101.30 | 2289.00 | 1281.00 |
| PG | **n** | 12 | 12 | 12 | 12 | 12 | 12 | 12 | 12 | 12 | 12 | 12 | 12 | 12 | 12 |
|  | **MD** | 69.30 | 4.03 | 4.40 | 38.33 | 317.10 | 111.80 | 127.00 | 412.80 | 83.78 | 233.50 | 6.86 | 30.91 | 3841.00 | 69.07 |
|  | **MIN** | 16.53 | 0.70 | 0.86 | 6.83 | 46.13 | 36.52 | 6.76 | 14.22 | 18.43 | 12.12 | 4.46 | 1.46 | 339.80 | 2.33 |
|  | **MAX** | 129.70 | 24.39 | 14.21 | 267.90 | 7321.00 | 564.60 | 448.70 | 3416.00 | 158.70 | 329.40 | 272.20 | 105.30 | 33200.00 | 1563.00 |
|  | **M** | 66.66 | 7.83 | 5.10 | 67.55 | 1030.00 | 165.70 | 129.60 | 632.90 | 85.20 | 191.80 | 29.85 | 40.10 | 8339.00 | 238.20 |
|  | **SD** | 37.46 | 8.14 | 3.76 | 77.65 | 2028.00 | 161.90 | 120.00 | 928.10 | 48.69 | 105.50 | 76.40 | 33.42 | 10061.00 | 442.50 |
| p-value | | **<0.0001**  **(*)** | **<0.0001**  **(*)** | **<0.0001**  **(*)** | **<0.0001**  **(*)** | **<0.0001**  **(*)** | **<0.0001**  **(*)** | **<0.0001**  **(*)** | **<0.0001**  **(*)** | **<0.0001**  **(*)** | **<0.0001**  **(*)** | **0.0018**  **(*)** | **<0.0001**  **(*)** | **0.0233**  **(*)** | 0.4347 |

|  |  | MCP-1 (CCL2) | MIP-1 alpha (CCL3) | MIP-1 beta (CCL4) | EGF | FGF-2 | PDGF-BB | VEGF-A |
| --- | --- | --- | --- | --- | --- | --- | --- | --- |
| AW | **n** | 10 | 10 | 10 | 10 | 10 | 10 | 10 |
|  | **MD** | 1396.00 | 25.52 | 146.50 | 8.71 | 41.18 | 47.29 | 3253.00 |
|  | **MIN** | 877.10 | 11.59 | 44.42 | 1.81 | 2.73 | 8.95 | 1076.00 |
|  | **MAX** | 5305.00 | 268.20 | 889.50 | 29.82 | 300.10 | 503.20 | 8969.00 |
|  | **M** | 1874.00 | 50.94 | 204.10 | 11.98 | 81.11 | 138.50 | 3571.00 |
|  | **SD** | 1373.00 | 77.03 | 245.60 | 9.21 | 96.71 | 175.40 | 2553.00 |
| WHD | **n** | 20 | 20 | 20 | 20 | 20 | 20 | 20 |
|  | **MD** | 724.00 | 238.40 | 445.70 | 1.81 | 14.55 | 24.62 | 1834.00 |
|  | **MIN** | 22.34 | 14.32 | 43.12 | 0.77 | 1.82 | 2.45 | 225.40 |
|  | **MAX** | 2005.00 | 1044.00 | 3327.00 | 34.81 | 936.00 | 260.70 | 22640.00 |
|  | **M** | 801.90 | 341.10 | 697.90 | 4.08 | 115.40 | 35.21 | 4483.00 |
|  | **SD** | 689.80 | 331.40 | 800.60 | 7.34 | 245.60 | 54.98 | 5611.00 |
| ALU | **n** | 15 | 15 | 15 | 15 | 15 | 15 | 15 |
|  | **MD** | 1367.00 | 402.10 | 719.00 | 9.39 | 174.50 | 44.89 | 3505.00 |
|  | **MIN** | 189.20 | 99.71 | 170.80 | 1.95 | 17.57 | 9.64 | 1113.00 |
|  | **MAX** | 5501.00 | 1876.00 | 3468.00 | 31.67 | 7026.00 | 306.80 | 7233.00 |
|  | **M** | 1410.00 | 522.50 | 1101.00 | 10.56 | 729.00 | 64.88 | 3401.00 |
|  | **SD** | 1335.00 | 459.60 | 1043.00 | 7.41 | 1786.00 | 72.59 | 1860.00 |
| VLU | **n** | 30 | 30 | 30 | 30 | 30 | 30 | 30 |
|  | **MD** | 1247.00 | 664.90 | 1730.00 | 5.19 | 155.40 | 37.57 | 6515.00 |
|  | **MIN** | 26.97 | 15.89 | 72.22 | 1.17 | 2.73 | 8.28 | 239.00 |
|  | **MAX** | 6040.00 | 2025.00 | 7986.00 | 17.36 | 8121.00 | 166.00 | 36575.00 |
|  | **M** | 1803.00 | 759.30 | 1781.00 | 6.04 | 698.70 | 46.13 | 7896.00 |
|  | **SD** | 1725.00 | 554.60 | 1621.00 | 3.98 | 1556.00 | 37.75 | 7187.00 |
| DFU | **n** | 15 | 15 | 15 | 15 | 15 | 15 | 15 |
|  | **MD** | 2226.00 | 324.80 | 293.50 | 8.91 | 414.70 | 101.90 | 2884.00 |
|  | **MIN** | 64.30 | 34.85 | 8.06 | 3.94 | 42.18 | 19.49 | 228.20 |
|  | **MAX** | 7256.00 | 5288.00 | 19735.00 | 66.12 | 1500.00 | 759.30 | 10774.00 |
|  | **M** | 2815.00 | 786.40 | 1883.00 | 15.76 | 513.00 | 173.40 | 3963.00 |
|  | **SD** | 2429.00 | 1322.00 | 5031.00 | 15.89 | 435.10 | 196.30 | 2976.00 |
| MIX | **n** | 8 | 8 | 8 | 8 | 8 | 8 | 8 |
|  | **MD** | 4224.00 | 2601.00 | 3611.00 | 12.97 | 442.10 | 81.37 | 7473.00 |
|  | **MIN** | 2008.00 | 258.20 | 110.50 | 6.54 | 22.29 | 51.82 | 3829.00 |
|  | **MAX** | 5715.00 | 3746.00 | 18880.00 | 18.67 | 1442.00 | 157.90 | 15825.00 |
|  | **M** | 4082.00 | 2228.00 | 5303.00 | 12.97 | 487.40 | 91.05 | 8478.00 |
|  | **SD** | 1508.00 | 1378.00 | 6201.00 | 4.11 | 443.40 | 39.58 | 4399.00 |
| PG | **n** | 12 | 12 | 12 | 12 | 12 | 12 | 12 |
|  | **MD** | 1350.00 | 294.40 | 836.40 | 6.93 | 121.40 | 40.36 | 5922.00 |
|  | **MIN** | 368.00 | 49.62 | 92.99 | 1.81 | 31.21 | 7.31 | 603.00 |
|  | **MAX** | 5877.00 | 1554.00 | 3776.00 | 20.81 | 1001.00 | 73.30 | 11595.00 |
|  | **M** | 1688.00 | 451.10 | 1100.00 | 8.18 | 275.40 | 41.15 | 6247.00 |
|  | **SD** | 1396.00 | 433.80 | 1054.00 | 5.56 | 313.30 | 22.38 | 3600.00 |
| p-value | | **0.0007**  **(*)** | **<0.0001**  **(*)** | **<0.0001**  **(*)** | **<0.0001**  **(*)** | **<0.0001**  **(*)** | **<0.0001**  **(*)** | **0.0037**  **(*)** |

1. Stage differentiation (INFE – infection, INFL – inflammation, PROL – proliferation, EPITH – epithelization)

|  |  | MMP-1 | MMP-2 | MMP-3 | MMP-7 | MMP-8 | MMP-9 | MMP-12 | MMP-13 | TIMP-1 | IL-1 alpha | IL-1  beta | IL-1RA | IL-4 | IL-6 |
| --- | --- | --- | --- | --- | --- | --- | --- | --- | --- | --- | --- | --- | --- | --- | --- |
| INFE | **n** | 13 | 13 | 13 | 13 | 13 | 13 | 13 | 13 | 13 | 13 | 13 | 13 | 13 | 13 |
|  | **MD** | 4944.00 | 48.26 | 237.80 | 175.50 | 33245.00 | 1340.00 | 117.40 | 59.09 | 1650.00 | 205.00 | 3825.00 | 61125.00 | 24.21 | 5883.00 |
|  | **MIN** | 247.20 | 5.95 | 27.76 | 23.42 | 8938.00 | 19.71 | 5.00 | 11.90 | 459.40 | 43.46 | 547.40 | 12535.00 | 8.22 | 529.00 |
|  | **MAX** | 40400.00 | 287.50 | 551.60 | 402.90 | 107620.00 | 8595.00 | 3212.00 | 709.50 | 15495.00 | 962.70 | 72075.00 | 797000.00 | 262.60 | 51100.00 |
|  | **M** | 13351.00 | 77.24 | 231.70 | 174.30 | 37604.00 | 2105.00 | 397.30 | 191.80 | 3428.00 | 261.50 | 10364.00 | 158292.00 | 68.37 | 9753.00 |
|  | **SD** | 16079.00 | 76.46 | 155.40 | 111.50 | 26096.00 | 2724.00 | 862.60 | 238.30 | 4307.00 | 248.10 | 19084.00 | 221067.00 | 86.47 | 13434.00 |
| INFLA | **n** | 41 | 41 | 41 | 41 | 41 | 41 | 41 | 41 | 41 | 41 | 41 | 41 | 41 | 41 |
|  | **MD** | 9175.00 | 86.19 | 152.20 | 231.20 | 26636.00 | 1386.00 | 352.70 | 43.47 | 2520.00 | 298.50 | 4332.00 | 150475.00 | 24.21 | 4964.00 |
|  | **MIN** | 602.30 | 3.27 | 15.20 | 21.61 | 2567.00 | 15.44 | 0.47 | 0.85 | 364.00 | 11.65 | 42.77 | 3105.00 | 7.07 | 77.09 |
|  | **MAX** | 64850.00 | 880.40 | 3664.00 | 6307.00 | 126200.00 | 130095.00 | 6158.00 | 3141.00 | 15270.00 | 4574.00 | 110350.00 | 1498500.00 | 486.40 | 135500.00 |
|  | **M** | 14166.00 | 141.60 | 364.90 | 544.20 | 32950.00 | 6203.00 | 981.10 | 318.70 | 3055.00 | 653.10 | 11901.00 | 226360.00 | 98.84 | 18545.00 |
|  | **SD** | 16152.00 | 177.20 | 630.40 | 1145.00 | 27914.00 | 20737.00 | 1646.00 | 685.70 | 2922.00 | 958.50 | 20225.00 | 291468.00 | 128.40 | 26941.00 |
| PROL | **n** | 40 | 40 | 40 | 40 | 40 | 40 | 40 | 40 | 40 | 40 | 40 | 40 | 40 | 40 |
|  | **MD** | 5007.00 | 33.61 | 143.60 | 226.80 | 20290.00 | 838.70 | 93.38 | 26.21 | 1693.00 | 106.50 | 1178.00 | 45245.00 | 14.05 | 4234.00 |
|  | **MIN** | 66.19 | 4.52 | 16.08 | 11.87 | 700.20 | 33.96 | 3.42 | 2.64 | 87.10 | 4.13 | 4.90 | 496.40 | 1.48 | 4.73 |
|  | **MAX** | 40400.00 | 226.60 | 1071.00 | 2493.00 | 124600.00 | 11007.00 | 5606.00 | 594.80 | 26450.00 | 1113.00 | 27885.00 | 1984500.00 | 303.80 | 104270.00 |
|  | **M** | 9024.00 | 51.73 | 204.90 | 418.30 | 29154.00 | 1195.00 | 485.10 | 75.25 | 5218.00 | 239.00 | 4054.00 | 145710.00 | 48.52 | 11212.00 |
|  | **SD** | 12559.00 | 56.41 | 210.90 | 552.00 | 28302.00 | 1789.00 | 1001.00 | 119.00 | 7287.00 | 278.60 | 6546.00 | 327046.00 | 70.73 | 19767.00 |
| EPITH | **n** | 16 | 16 | 16 | 16 | 16 | 16 | 16 | 16 | 16 | 16 | 16 | 16 | 16 | 16 |
|  | **MD** | 4118.00 | 8.43 | 57.66 | 387.30 | 21428.00 | 339.60 | 267.90 | 11.65 | 1892.00 | 207.40 | 2177.00 | 202175.00 | 12.65 | 1151.00 |
|  | **MIN** | 191.20 | 7.94 | 9.96 | 56.27 | 6472.00 | 18.35 | 4.20 | 3.39 | 62.40 | 9.29 | 53.25 | 10114.00 | 7.07 | 9.18 |
|  | **MAX** | 40400.00 | 598.20 | 504.80 | 17555.0 | 126200.00 | 13375.00 | 2120.00 | 1152.00 | 10701.00 | 682.90 | 36995.00 | 8002000.00 | 236.60 | 51100.00 |
|  | **M** | 11498.00 | 68.06 | 160.60 | 1429.00 | 31329.00 | 2166.00 | 609.70 | 114.00 | 2627.00 | 212.00 | 4590.00 | 1295201.00 | 33.73 | 9806.00 |
|  | **SD** | 15429.00 | 148.00 | 181.50 | 4308.00 | 30435.00 | 3810.00 | 751.60 | 287.60 | 3023.00 | 191.90 | 9188.00 | 2688280.00 | 57.81 | 17608.00 |
| p-value | | 0.2868 | **0.0175 (*)** | 0.2367 | 0.1778 | 0.2696 | 0.1374 | 0.1029 | **0.0381**  **(*)** | 0.6351 | **0.0111**  **(*)** | **0.0035**  **(*)** | **0.0057**  **(*)** | **0.0366 (*)** | 0.1573 |

|  |  | IL-9 | IL-10 | IL-13 | IL-17A | IL-18 | IL-21 | IFN gamma | G-CSF | GM-CSF | TNF alpha | TNF  beta | TGF alpha | IL-8 (CXCL8) | IP-10 (CXCL10) |
| --- | --- | --- | --- | --- | --- | --- | --- | --- | --- | --- | --- | --- | --- | --- | --- |
| INFE | **n** | 13 | 13 | 13 | 13 | 13 | 13 | 13 | 13 | 13 | 13 | 13 | 13 | 13 | 13 |
|  | **MD** | 78.12 | 6.81 | 4.40 | 31.30 | 316.30 | 107.60 | 75.12 | 337.00 | 44.23 | 235.50 | 6.86 | 15.02 | 7153.00 | 19.41 |
|  | **MIN** | 15.05 | 0.46 | 1.63 | 7.19 | 116.10 | 44.10 | 17.14 | 30.79 | 16.87 | 55.35 | 6.13 | 1.38 | 1324.00 | 2.37 |
|  | **MAX** | 129.70 | 41.62 | 14.81 | 193.00 | 2278.00 | 515.70 | 225.50 | 3312.00 | 158.70 | 422.90 | 371.90 | 73.48 | 67310.00 | 372.20 |
|  | **M** | 78.44 | 10.85 | 6.03 | 45.57 | 507.20 | 199.50 | 85.15 | 675.80 | 57.68 | 210.80 | 35.83 | 25.05 | 15694.00 | 52.82 |
|  | **SD** | 34.61 | 11.65 | 4.26 | 49.98 | 570.60 | 175.90 | 59.70 | 972.40 | 42.82 | 95.96 | 101.00 | 22.41 | 18934.00 | 99.73 |
| INFLA | **n** | 41 | 41 | 41 | 41 | 41 | 41 | 41 | 41 | 41 | 41 | 41 | 41 | 41 | 41 |
|  | **MD** | 69.42 | 9.43 | 4.40 | 39.76 | 416.30 | 109.50 | 130.50 | 354.50 | 71.72 | 268.80 | 6.86 | 25.19 | 3961.00 | 59.77 |
|  | **MIN** | 7.88 | 0.70 | 0.60 | 2.26 | 65.39 | 15.23 | 6.76 | 16.11 | 8.07 | 12.12 | 4.46 | 3.10 | 339.80 | 2.33 |
|  | **MAX** | 131.50 | 303.70 | 38.17 | 571.10 | 12415.00 | 1426.00 | 891.30 | 6548.00 | 876.00 | 1443.00 | 2734.00 | 343.20 | 32810.00 | 3805.00 |
|  | **M** | 67.07 | 24.00 | 8.56 | 109.20 | 1175.00 | 279.20 | 202.60 | 1107.00 | 132.10 | 337.90 | 120.80 | 50.22 | 6306.00 | 299.00 |
|  | **SD** | 32.54 | 48.81 | 9.13 | 144.00 | 2240.00 | 340.50 | 204.90 | 1523.00 | 173.60 | 328.00 | 443.20 | 62.51 | 7318.00 | 668.20 |
| PROL | **n** | 40 | 40 | 40 | 40 | 40 | 40 | 40 | 40 | 40 | 40 | 40 | 40 | 40 | 40 |
|  | **MD** | 52.27 | 3.33 | 4.40 | 19.91 | 173.50 | 92.14 | 51.12 | 70.58 | 45.02 | 144.40 | 6.86 | 12.95 | 3061.00 | 49.90 |
|  | **MIN** | 5.66 | 0.21 | 0.33 | 2.26 | 24.25 | 16.89 | 7.18 | 7.43 | 5.80 | 2.58 | 2.58 | 0.69 | 407.60 | 1.67 |
|  | **MAX** | 108.60 | 93.84 | 21.05 | 297.50 | 1199.00 | 1005.00 | 439.10 | 4414.00 | 313.50 | 835.40 | 272.20 | 133.40 | 37995.00 | 1563.00 |
|  | **M** | 53.70 | 13.61 | 5.08 | 51.13 | 244.50 | 169.70 | 79.02 | 602.70 | 62.02 | 180.50 | 15.88 | 22.92 | 4616.00 | 191.60 |
|  | **SD** | 24.25 | 21.41 | 4.50 | 69.42 | 264.30 | 194.00 | 81.57 | 1187.00 | 65.57 | 153.50 | 42.81 | 28.35 | 6054.00 | 316.80 |
| EPITH | **n** | 16 | 16 | 16 | 16 | 16 | 16 | 16 | 16 | 16 | 16 | 16 | 16 | 16 | 16 |
|  | **MD** | 36.79 | 3.02 | 4.40 | 44.47 | 209.30 | 66.71 | 43.08 | 91.05 | 18.43 | 50.10 | 6.86 | 38.50 | 2811.00 | 82.21 |
|  | **MIN** | 13.11 | 0.31 | 0.86 | 1.53 | 13.03 | 26.30 | 9.78 | 10.00 | 13.01 | 5.10 | 6.13 | 1.01 | 875.60 | 3.12 |
|  | **MAX** | 103.20 | 42.04 | 14.21 | 1476.00 | 1252.00 | 698.30 | 422.50 | 3460.00 | 141.90 | 312.10 | 39.68 | 261.50 | 77120.00 | 663.60 |
|  | **M** | 49.61 | 8.49 | 4.71 | 146.10 | 317.40 | 119.30 | 82.91 | 534.10 | 52.95 | 115.40 | 8.87 | 59.18 | 13398.00 | 182.20 |
|  | **SD** | 29.13 | 12.15 | 3.24 | 358.90 | 366.70 | 166.60 | 104.80 | 936.80 | 46.53 | 104.80 | 8.22 | 74.55 | 21457.00 | 223.90 |
| p-value | | **0.0271**  **(*)** | 0.0684 | 0.4521 | 0.1000 | **0.0009**  **(*)** | 0.061 | **0.0079**  **(*)** | **0.0145**  **(*)** | **0.0084**  **(*)** | **0.0041**  **(*)** | 0.9275 | 0.0512 | 0.0996 | 0.1581 |

|  |  | MCP-1 (CCL2) | MIP-1 alpha (CCL3) | MIP-1 beta (CCL4) | EGF | FGF-2 | PDGF-BB | VEGF-A |
| --- | --- | --- | --- | --- | --- | --- | --- | --- |
| INFE | **n** | 13 | 13 | 13 | 13 | 13 | 13 | 13 |
|  | **MD** | 873.10 | 300.00 | 671.90 | 7.27 | 188.40 | 44.89 | 3677.00 |
|  | **MIN** | 86.28 | 91.32 | 138.00 | 2.90 | 42.18 | 19.49 | 1224.00 |
|  | **MAX** | 5501.00 | 1204.00 | 2571.00 | 16.05 | 2883.00 | 152.10 | 11525.00 |
|  | **M** | 1250.00 | 485.70 | 908.80 | 8.33 | 628.30 | 57.03 | 4725.00 |
|  | **SD** | 1460.00 | 382.30 | 820.80 | 3.65 | 930.30 | 33.71 | 2974.00 |
| INFLA | **n** | 41 | 41 | 41 | 41 | 41 | 41 | 41 |
|  | **MD** | 1718.00 | 651.90 | 1575.00 | 8.75 | 265.50 | 55.24 | 5612.00 |
|  | **MIN** | 22.34 | 34.85 | 8.06 | 1.63 | 2.73 | 7.31 | 228.20 |
|  | **MAX** | 7256.00 | 5288.00 | 19735.00 | 66.12 | 7026.00 | 759.30 | 17805.00 |
|  | **M** | 2534.00 | 1034.00 | 2558.00 | 10.74 | 517.90 | 85.43 | 6947.00 |
|  | **SD** | 2063.00 | 1173.00 | 4214.00 | 10.68 | 1103.00 | 125.40 | 4681.00 |
| PROL | **n** | 40 | 40 | 40 | 40 | 40 | 40 | 40 |
|  | **MD** | 1569.00 | 280.40 | 506.70 | 5.28 | 71.56 | 29.65 | 3429.00 |
|  | **MIN** | 26.97 | 11.59 | 43.12 | 0.99 | 2.73 | 2.45 | 225.40 |
|  | **MAX** | 5877.00 | 2207.00 | 7986.00 | 31.67 | 1625.00 | 503.20 | 13475.00 |
|  | **M** | 1829.00 | 458.90 | 1047.00 | 8.13 | 245.30 | 77.15 | 4259.00 |
|  | **SD** | 1526.00 | 552.50 | 1595.00 | 7.94 | 378.50 | 116.50 | 3335.00 |
| EPITH | **n** | 16 | 16 | 16 | 16 | 16 | 16 | 16 |
|  | **MD** | 507.00 | 268.10 | 487.20 | 3.05 | 24.16 | 26.93 | 2432.00 |
|  | **MIN** | 41.95 | 15.89 | 64.22 | 0.77 | 1.82 | 6.36 | 239.00 |
|  | **MAX** | 2226.00 | 1591.00 | 2457.00 | 34.81 | 8121.00 | 260.70 | 36575.00 |
|  | **M** | 739.30 | 370.70 | 702.70 | 6.59 | 668.60 | 58.68 | 6180.00 |
|  | **SD** | 632.70 | 432.30 | 718.40 | 9.20 | 2009.00 | 72.64 | 9782.00 |
| p-value | | **0.0009**  **(*)** | **0.0073**  **(*)** | **0.0471**  **(*)** | **0.0164 (*)** | **0.0049 (*)** | 0.1852 | **0.0192**  **(*)** |

1. Infection status (N-INF – non-infected, COL – colonized, INF – infected)

|  |  | MMP-1 | MMP-2 | MMP-3 | MMP-7 | MMP-8 | MMP-9 | MMP-12 | MMP-13 | TIMP-1 | IL-1 alpha | IL-1  beta | IL-1RA | IL-4 | IL-6 |
| --- | --- | --- | --- | --- | --- | --- | --- | --- | --- | --- | --- | --- | --- | --- | --- |
| N-INF | **n** | 40 | 40 | 40 | 40 | 40 | 40 | 40 | 40 | 40 | 40 | 40 | 40 | 40 | 40 |
|  | **MD** | 2792.00 | 44.17 | 201.30 | 276.20 | 18280.00 | 926.20 | 95.69 | 21.83 | 2964.00 | 199.40 | 1848.00 | 82245.00 | 12.65 | 3478.00 |
|  | **MIN** | 191.20 | 5.43 | 13.37 | 11.87 | 700.20 | 18.35 | 4.20 | 3.39 | 62.40 | 5.70 | 4.90 | 358.90 | 1.48 | 45.73 |
|  | **MAX** | 40400.00 | 164.20 | 1071.00 | 17555.00 | 126200.00 | 13375.00 | 4223.00 | 2730.00 | 26450.00 | 3204.00 | 17735.00 | 8002000.00 | 486.40 | 135500.00 |
|  | **M** | 9908.00 | 52.10 | 244.40 | 925.70 | 24877.00 | 1852.00 | 477.00 | 183.00 | 6071.00 | 262.30 | 2967.00 | 528068.00 | 56.81 | 14089.00 |
|  | **SD** | 13814.00 | 46.33 | 226.80 | 2812.00 | 23129.00 | 3104.00 | 859.90 | 468.00 | 7258.00 | 504.50 | 4232.00 | 1659626.00 | 95.21 | 28239.00 |
| COL | **n** | 57 | 57 | 57 | 57 | 57 | 57 | 57 | 57 | 57 | 57 | 57 | 57 | 57 | 57 |
|  | **MD** | 7046.00 | 46.07 | 133.90 | 228.90 | 26490.00 | 973.50 | 281.80 | 34.54 | 1698.00 | 273.70 | 4063.00 | 110395.00 | 23.08 | 4820.00 |
|  | **MIN** | 66.19 | 3.27 | 9.96 | 21.61 | 2567.00 | 15.44 | 0.47 | 0.85 | 87.10 | 4.13 | 38.27 | 642.30 | 2.64 | 4.73 |
|  | **MAX** | 64850.00 | 880.40 | 3664.00 | 6307.00 | 126200.00 | 130095.00 | 6158.00 | 3141.00 | 15270.00 | 4574.00 | 110350.00 | 1984500.00 | 405.50 | 59435.00 |
|  | **M** | 12797.00 | 120.70 | 279.80 | 436.30 | 35496.00 | 4608.00 | 882.50 | 185.60 | 2336.00 | 512.90 | 10612.00 | 212398.00 | 74.74 | 14072.00 |
|  | **SD** | 15269.00 | 173.70 | 548.00 | 854.20 | 30787.00 | 17692.00 | 1514.00 | 488.30 | 2285.00 | 769.40 | 18240.00 | 349481.00 | 104.90 | 18546.00 |
| INF | **n** | 13 | 13 | 13 | 13 | 13 | 13 | 13 | 13 | 13 | 13 | 13 | 13 | 13 | 13 |
|  | **MD** | 4944.00 | 48.26 | 237.80 | 175.50 | 33245.00 | 1340.00 | 117.40 | 59.09 | 1650.00 | 205.00 | 3825.00 | 61125.00 | 24.21 | 5883.00 |
|  | **MIN** | 247.20 | 5.95 | 27.76 | 23.42 | 8938.00 | 19.71 | 5.00 | 11.90 | 459.40 | 43.46 | 547.40 | 12535.00 | 8.22 | 529.00 |
|  | **MAX** | 40400.00 | 287.50 | 551.60 | 402.90 | 107620.00 | 8595.00 | 3212.00 | 709.50 | 15495.00 | 962.70 | 72075.00 | 797000.00 | 262.60 | 51100.00 |
|  | **M** | 13351.00 | 77.24 | 231.70 | 174.30 | 37604.00 | 2105.00 | 397.30 | 191.80 | 3428.00 | 261.50 | 10364.00 | 158292.00 | 68.37 | 9753.00 |
|  | **SD** | 16079.00 | 76.46 | 155.40 | 111.50 | 26096.00 | 2724.00 | 862.60 | 238.30 | 4307.00 | 248.10 | 19084.00 | 221067.00 | 86.47 | 13434.00 |
| p-value | | 0.2269 | 0.5190 | 0.2986 | 0.0806 | 0.0515 | 0.8144 | 0.0846 | 0.2015 | 0.1315 | **0.0190**  **(*)** | **0.0043**  **(*)** | 0.7385 | 0.1586 | 0.7447 |

|  |  | IL-9 | IL-10 | IL-13 | IL-17A | IL-18 | IL-21 | IFN gamma | G-CSF | GM-CSF | TNF alpha | TNF  beta | TGF alpha | IL-8 (CXCL8) | IP-10 (CXCL10) |
| --- | --- | --- | --- | --- | --- | --- | --- | --- | --- | --- | --- | --- | --- | --- | --- |
| N-INF | **n** | 40 | 40 | 40 | 40 | 40 | 40 | 40 | 40 | 40 | 40 | 40 | 40 | 40 | 40 |
|  | **MD** | 48.60 | 3.58 | 4.40 | 23.10 | 190.20 | 80.94 | 49.05 | 75.37 | 48.48 | 161.30 | 6.86 | 14.42 | 2877.00 | 85.12 |
|  | **MIN** | 7.88 | 0.49 | 0.33 | 1.53 | 13.03 | 26.30 | 6.76 | 10.00 | 8.07 | 5.10 | 2.58 | 1.01 | 407.60 | 3.12 |
|  | **MAX** | 131.50 | 93.84 | 38.17 | 1476.00 | 1202.00 | 1092.00 | 705.70 | 6548.00 | 313.50 | 1365.00 | 384.30 | 261.50 | 12640000.00 | 1550.00 |
|  | **M** | 53.51 | 14.48 | 5.75 | 91.59 | 267.70 | 173.30 | 83.26 | 708.40 | 61.01 | 188.50 | 18.07 | 32.63 | 322255.00 | 238.90 |
|  | **SD** | 30.02 | 22.04 | 6.41 | 239.90 | 274.10 | 215.20 | 114.70 | 1359.00 | 64.13 | 215.50 | 60.07 | 47.49 | 1997571.00 | 338.10 |
| COL | **n** | 57 | 57 | 57 | 57 | 57 | 57 | 57 | 57 | 57 | 57 | 57 | 57 | 57 | 57 |
|  | **MD** | 64.12 | 5.98 | 4.40 | 36.90 | 281.50 | 109.30 | 111.70 | 159.60 | 69.26 | 195.80 | 6.86 | 24.02 | 3570.00 | 45.50 |
|  | **MIN** | 5.66 | 0.21 | 0.60 | 2.26 | 24.25 | 15.23 | 6.76 | 7.43 | 5.80 | 2.58 | 4.46 | 0.69 | 339.80 | 1.67 |
|  | **MAX** | 122.30 | 303.70 | 33.49 | 571.10 | 12415.00 | 1426.00 | 891.30 | 6034.00 | 876.00 | 1443.00 | 2734.00 | 343.20 | 77120.00 | 3805.00 |
|  | **M** | 62.31 | 19.04 | 7.01 | 91.14 | 917.70 | 231.80 | 166.00 | 871.90 | 110.60 | 269.80 | 87.81 | 45.92 | 7037.00 | 233.10 |
|  | **SD** | 28.74 | 42.17 | 7.26 | 121.80 | 1947.00 | 299.60 | 178.50 | 1300.00 | 153.20 | 273.90 | 376.70 | 59.87 | 11413.00 | 572.50 |
| INF | **n** | 13 | 13 | 13 | 13 | 13 | 13 | 13 | 13 | 13 | 13 | 13 | 13 | 13 | 13 |
|  | **MD** | 78.12 | 6.81 | 4.40 | 31.30 | 316.30 | 107.60 | 75.12 | 337.00 | 44.23 | 235.50 | 6.86 | 15.02 | 7153.00 | 19.41 |
|  | **MIN** | 1.00 | 0.46 | 1.63 | 7.19 | 116.10 | 44.10 | 17.14 | 30.79 | 16.87 | 55.35 | 6.13 | 1.38 | 1324.00 | 2.37 |
|  | **MAX** | 129.70 | 41.62 | 14.81 | 193.00 | 2278.00 | 515.70 | 225.50 | 3312.00 | 158.70 | 422.90 | 371.90 | 73.48 | 67310.00 | 372.20 |
|  | **M** | 75.43 | 10.85 | 6.03 | 45.57 | 507.20 | 199.50 | 85.15 | 675.80 | 57.68 | 210.80 | 35.83 | 25.05 | 15694.00 | 52.81 |
|  | **SD** | 39.57 | 11.65 | 4.26 | 49.98 | 570.60 | 175.90 | 59.70 | 972.40 | 42.82 | 95.96 | 101.00 | 22.41 | 18934.00 | 99.73 |
| p-value | | 0.0687 | 0.6868 | 0.8638 | 0.2108 | **0.0264**  **(*)** | 0.214 | 0.0571 | 0.1754 | 0.0573 | 0.3048 | 0.4078 | 0.3864 | 0.088 | **0.0182**  **(*)** |

|  |  | MCP-1 (CCL2) | MIP-1 alpha (CCL3) | MIP-1 beta (CCL4) | EGF | FGF-2 | PDGF-BB | VEGF-A |
| --- | --- | --- | --- | --- | --- | --- | --- | --- |
| N-INF | **n** | 40 | 40 | 40 | 40 | 40 | 40 | 40 |
|  | **MD** | 1284.00 | 306.40 | 487.20 | 4.87 | 75.47 | 31.53 | 4085.00 |
|  | **MIN** | 41.95 | 11.59 | 44.42 | 0.77 | 1.82 | 8.28 | 239.00 |
|  | **MAX** | 7256.00 | 5288.00 | 19735.00 | 66.12 | 8121.00 | 759.30 | 22640.00 |
|  | **M** | 1711.00 | 556.70 | 1373.00 | 9.06 | 590.40 | 92.72 | 4642.00 |
|  | **SD** | 1657.00 | 900.60 | 3168.00 | 12.21 | 1665.00 | 152.30 | 4114.00 |
| COL | **n** | 57 | 57 | 57 | 57 | 57 | 57 | 57 |
|  | **MD** | 1638.00 | 400.20 | 1001.00 | 6.66 | 174.50 | 48.33 | 4319.00 |
|  | **MIN** | 22.34 | 14.32 | 8.06 | 0.99 | 2.73 | 2.45 | 225.40 |
|  | **MAX** | 6040.00 | 3746.00 | 18880.00 | 31.67 | 1625.00 | 444.60 | 36575.00 |
|  | **M** | 2113.00 | 779.00 | 1809.00 | 8.92 | 318.10 | 67.00 | 6463.00 |
|  | **SD** | 1862.00 | 902.40 | 2940.00 | 6.97 | 367.50 | 76.21 | 6208.00 |
| INF | **n** | 13 | 13 | 13 | 13 | 13 | 13 | 13 |
|  | **MD** | 873.10 | 300.00 | 671.90 | 7.27 | 188.40 | 44.89 | 3677.00 |
|  | **MIN** | 86.28 | 91.32 | 138.00 | 2.90 | 42.18 | 19.49 | 1224.00 |
|  | **MAX** | 5501.00 | 1204.00 | 2571.00 | 16.05 | 2883.00 | 152.10 | 11525.00 |
|  | **M** | 1250.00 | 485.70 | 908.80 | 8.33 | 628.30 | 57.03 | 4725.00 |
|  | **SD** | 1460.00 | 382.30 | 820.80 | 3.65 | 930.30 | 33.71 | 2974.00 |
| p-value | | 0.1233 | 0.1602 | 0.3343 | 0.2467 | 0.0842 | 0.671 | 0.3965 |
